# Adverse events of anti-IL-5 drugs in patients with eosinophilic asthma: a meta-analysis of randomized controlled trials and real-world evidence-based assessments

**DOI:** 10.1186/s12890-024-02885-2

**Published:** 2024-02-03

**Authors:** Wen Li, Shi-Chao Tang, Lei Jin

**Affiliations:** 1 Shanghai Jiao Tong University School of Medicine, Shanghai, 200025 China; 2 Department of Rheumatology and Immunology, Tongren Hospital, Shanghai Jiao Tong University School of Medicine, Shanghai, 200336 China; 3 Department of Rheumatology, Immunology & Allergy, Shanghai General Hospital, Shanghai Jiao Tong University School of Medicine, Shanghai, 200080 China

**Keywords:** Mepolizumab, Benralizumab, Reslizumab, Anti-interleukin-5, Adverse events, Eosinophilic asthma

## Abstract

**Background:**

We aimed to clarify comprehensively the safety profiles of anti-IL-5 drugs and pinpoint potential safety concerns that may arise in their post-marketing phase.

**Methods:**

Two researchers conducted comprehensive searches of PubMed, EMBASE, Web of Science, and the Cochrane Library from inception to September 2022. Additionally, we investigated the FDA AE Reporting System for post-marketing adverse event (AE) reports related to anti-IL-5 drugs. The outcomes fulfilled the proportional reporting rate criteria and the Bayesian confidence propagation neural network.

**Results:**

We included 24 published studies in our analysis. The anti-IL-5 treatment group showed an incidence of AEs comparable to the placebo group, and it exhibited a significantly lower frequency of serious AEs. Common AEs were asthma, nasopharyngitis, headache, upper respiratory tract infection (URTI), and bronchitis. The post-marketing data included 28,478 case reports associated with the suspect drugs and 75 suspect safety observations affecting 16 system organ classes. New suspect observations included incomplete therapeutic product effect, URTIs, and pulmonary mass in reports related to mepolizumab. Reports associated with mepolizumab and benralizumab also indicated issues with incorrect technique in device usage and product issues.

**Conclusions:**

Individual anti-IL-5 drugs’ safety profiles largely matched their product inserts. We identified issues like improper device usage, product issue, and URTIs as potential concerns for mepolizumab and benralizumab. Additionally, all anti-IL-5 drugs showed signs of incomplete therapeutic effects.

**Supplementary Information:**

The online version contains supplementary material available at 10.1186/s12890-024-02885-2.

## Background

Eosinophilic asthma represents a prevalent, chronic respiratory condition characterized by airflow obstruction, bronchial inflammation, and heightened airway responsiveness. Its pathogenesis often involves airway inflammation, predominantly seen as eosinophilic infiltration, as evidenced by consistent blood eosinophils presence [1–3]. Eosinophilic granular release-induced tissue damage drives inflammation progression and airway remodeling [4, 5]. The Global Burden of Disease Study 2019 estimated that approximately 262 million individuals worldwide suffer from eosinophilic asthma, leading to around 461,000 fatalities annually [6, 7]. Presently, the primary treatments include inhaled corticosteroids (ICS), oral corticosteroids (OCS), short-acting β2 agonists, long-acting β2 agonists (LABA) and long-acting muscarinic receptor antagonist (LAMA). While conventional treatments effectively manage asthma in many instances, they often exacerbate the overall disease burden due to potential adverse effects. In contrast, additional treatments such as antileukotrienes and cromones offer limited clinical benefits or are prohibitively expensive [8]. To this end, the 2022 GINA guidelines endorse the usage of biologic therapies as adjunctive therapies for managing severe eosinophilic asthma effectively [9].

Biologic therapies,including anti-immunoglobulin E (anti-IgE) (omalizumab), anti-IL-4Rα (dupilumab), anti-thymic stomal lymphopoietin (anti-TSLP) (tezepelumab), play a pivotal role in the management of eosinophilic asthma. Studies on omalizumab indicated for asthma showed overall incidence of adverse events is similar to that of the placebo-controlled groups [10, 11]. The phase III clinical trial “Efficacy and Safety of Dupilumab in Glucocorticoid-Dependent Severe Asthma” reported similar adverse event rates in both the dupilumab (62%) and placebo (64%) groups [12]. Additionally, in the phase IIa clinical study “Dupilumab in Persistent Asthma with Elevated Eosinophil Levels,” adverse events were reported by comparable proportions of patients in both groups (77% in the placebo group versus 81% in the dupilumab group) [13]. The phase 2b Pathway study and phase 3 Navigator trial, evaluating tezepelumab for severe asthma treatment, also showed similar adverse event incidences between treatment groups, with 75% (*n* = 496 of 665) in the tezepelumab group and 77% (*n* = 512 of 669) in the placebo group [14, 15]. These findings underscore the clinical value of biologic drugs in managing eosinophilic asthma.

Anti-IL-5 drugs (mepolizumab, reslizumab, and benralizumab), which are also grouped in the scope of respiratory biologic treatments and recommended by the BTS/SIGN British Guidelines for managing severe eosinophilic asthma, are now widely used clinically in Europe [16]. Clinical trials highlighted their efficacy, particularly in reducing sputum eosinophils and enhancing airway function. Mepolizumab, benralizumab, and reslizumab have maintained a consistent safety profile across nearly all randomized placebo-controlled trials, with AE rates similar to those in placebo groups [17–40]. The Product inserts for mepolizumab and benralizumab list risks including headache, hypersensitivity reactions, infections, injection site reactions and pyrexia, reslizumab shares the risk of hypersensitivity reactions in common. Besides, risks such as back pain, nasal congestion, abdominal pain, increased blood creatine phosphokinase and myalgia are unique to either mepolizumab or reslizumab [41–43]. Mepolizumab, benralizumab, and reslizumab, while all targeting IL-5, exhibit unique yet overlapping safety profiles. Given their limited duration on the market, a comprehensive analysis of safety data from clinical trials and post-marketing experiences is essential. These anti-IL-5 drugs have not yet been used clinically for asthma treatment in China. Consequently, this study aims to elucidate the overall safety profile of anti-IL-5 treatments, highlight similarities and differences in their safety profiles, and identify potential novel safety signals to provide a more robust safety reference for future clinical use.

## Methods

### Analysis of adverse events from clinical trials in published papers (meta-analysis)

This analysis of adverse events (AEs) associated with anti-IL-5 treatments (mepolizumab, benralizumab, reslizumab) in clinical trials adheres to guidelines of the Preferred Reporting Items for Systematic Reviews and Meta-Analyses [44].

### Search strategy

Two researchers independently conducted thorough searches in PubMed, EMBASE, Web of Science, and the Cochrane Library for articles published from the earliest available date up to September 2022. A combination of MeSH terms and free text terms was hereby used (Appendix 1), involving terms such as “mepolizumab”, “Bosatria”, “SB-240563”, “SB240563”, “Nucala”, “reslizumab”, “SCH-55700”, “SCH55700”, “SCH 55700”, “CEP-38072”, “CEP38072”, “Cinqair”, “DCP-835”, “DCP835”, “DCP 835”, “benralizumab”, “MEDI-563”, “MEDI 563”, “Fasenra”, “BIW-8405”, “asthma”, “asthmas”, “bronchial asthma”, and “asthma, bronchial”. The literature search was unrestricted in terms of study design, publication type, or language. Search results were imported into EndNoteX9 for article evaluation and selection. Disagreement about article triage was settled through discussion between researchers.

### Inclusion & exclusion criteria

We included studies that were randomized placebo-controlled trials, involved patients with eosinophilic asthma, used mepolizumab, benralizumab, or reslizumab regardless of dosage or administration methods, and focused on AEs as defined in the “International Council for Harmonisation of Technical Requirements for Pharmaceuticals for Human Use Good Clinical Practice (ICH GCP)” [45].

We excluded studies that weren’t original research, like review articles, case reports, conference abstracts, corrections, comments, letters, editorials, notes, book chapters, surveys, consensus documents, guidelines, trial registry records, and protocols; studies that didn’t meet our criteria for interventions, design, or target population, such as non-randomized trials, randomized trials with active control, animal studies, pharmacokinetic studies, or post hoc analyses; studies where we couldn’t access the full text; studies lacking clear results on AEs or those unsuitable for statistical analysis; and studies not written in English.

### Data extraction

Herein, the preliminary literature screening was independently performed via analysis of article titles and abstracts, strictly adhering to the inclusion and exclusion criteria. Post-identification in the preliminary screening, full texts and supplementary appendices was individually assessed, and corresponding data were extracted. The extracted data encompassed descriptive elements from all included studies, such as the first author, publication year, study type, patient characteristics, interventions, and follow-up duration. Discrepancies in the extracted data were resolved through dialogues between reviewers.

### Risk of bias assessment

Utilizing the Cochrane Collaboration’s tool [46], an independent risk of bias assessment was conducted by expert reviewers. Low, unclear, or high-risk biases were ascertained based on evaluation of random sequence generation, allocation concealment, blinding of participants and personnel, blinding of outcome assessment, incomplete outcome data, selective reporting, and other bias methods for each RCT.

### Data analysis

Risk ratios (RRs) and 95% confidence intervals (CIs) were calculated for all dichotomous AE outcomes within this meta-analysis, and the heterogeneity between studies was assessed using both the *P* value and the I^2^ statistic. This evaluation was performed both for each sub-treatment group and the overall treatment group using the I^2^ statistic, with a P value less than 0.05 or an I^2^ value equal to or above 50% in the Cochrane Q test indicating significant heterogeneity. A fixed-effect model was utilized unless the I^2^ value reached or exceeded 50%, in which case, a random-effects model was applied. Meanwhile, subgroup analysis for anti-IL-5 treatments and post hoc sensitivity analysis were executed, the latter involving a switch from a random effect model to a fixed effect model to identify the source of heterogeneity. If the source of heterogeneity was suspected by several factors, a meta-regression was performed to explore factors contributing to heterogeneity. When at least 10 studies were involved in an outcome, a funnel plot was generated for the evaluation of potential publication bias, with Egger’s test applied to quantify the significance of said bias. Dichotomous data were analysed using Stata version 15.1 (StataCorp LLC, U.S.A.), and descriptive statistics were calculated using Microsoft Excel 2013 (Microsoft Corporation, U.S.A.). Besides, the risk of bias assessment was evaluated using Review Manager (RevMan) software version 5.4.1 (The Cochrane Collaboration, 2020).

## Analysis of adverse events from post-marketing reports (PRR and BNCPP)

### Data source

Post-marketing adverse event data for anti-IL-5 drugs (mepolizumab, benralizumab, and reslizumab) were gleaned from the FDA Adverse Event Reporting System (FAERS). Employed in safety surveillance and on-market product evaluation, and provided by healthcare professionals, consumers, and manufacturing authorization holders globally, FAERS aggregated spontaneous post-marketing adverse event data, as well as reports from studies and programs after marketing, irrespective of whether these events were transpired within or beyond U.S. borders. Herein, all reported AEs were codified with Preferred Terms (PTs) according to the Medical Dictionary for Drug Regulatory Activities to facilitate subsequent statistical evaluation.

### Data extraction and process

From January 1, 2018, to September 30, 2022, a conjunction of keywords (“mepolizumab,” “Nucala,” “reslizumab,” “Cinqair,” “Fasenra,” “benralizumab”) was deployed to search FAERS, yielding adverse event data related to suspect anti-IL-5 drugs. The top 100 AEs at a PT level, ranked by the number of cases, were then identified through signal detection.

### Signal detection and evaluation

Herein, the Proportional Reporting Rate (PRR) and the Bayesian Confidence Propagation Neural Network (BCPNN) were utilized to identify potential safety observations among the top 100 AEs at a PT level [47–49]. The former is based on the premise that a specific adverse event connected to a certain medicinal product is reported statistically more often in association with that product than with others, while the latter integrates the principle of ratio imbalance and the Bayesian approach to facilitate signal detection. Both methodologies were hereby implemented using data from FAERS, with 2 × 2 contingency tables and algorithms employed for signal detection (Appendices 2 & 3). Besides, safety observations suspected on the basis of both PRR and BCPNN were further examined using clinical judgement to discern potential new safety signals and illustrate the comprehensive safety profile of anti-IL-5 drugs.

## Results

### Outcomes from adverse event analysis in clinical trials via published studies (meta-analysis)

#### Search results

**Fig. 1:**
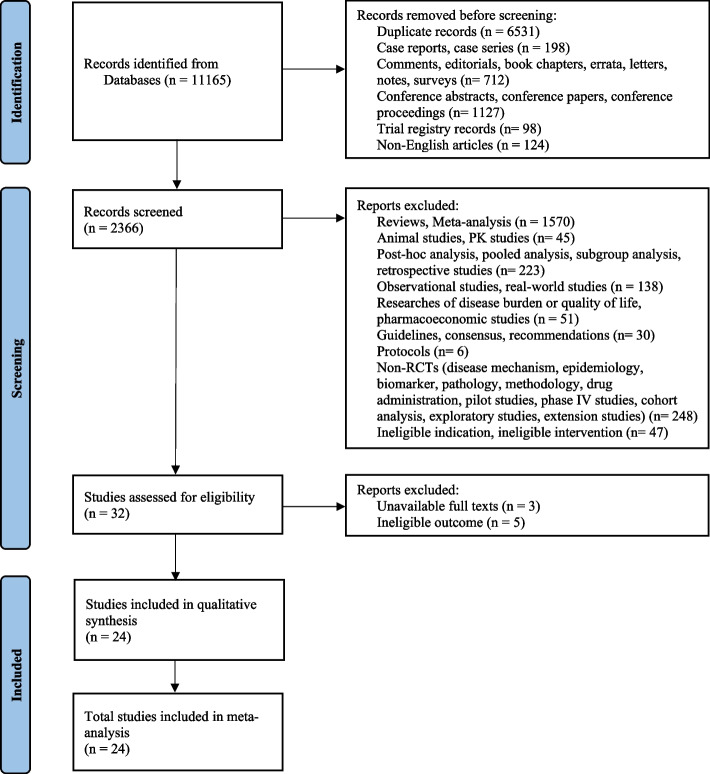
Flow chart of study selection

An extensive search yielded 11,165 articles across PubMed (*n* = 1711), Cochrane (*n* = 559), EMBASE (*n* = 6011), and Web of Science (*n* = 2884). Upon preliminary appraisal, 32 of them merited comprehensive evaluation. However, 8 were disqualified due to inaccessible full text and ineligible adverse event outcomes. Consequently, 24 encompassing randomized, placebo-controlled trials were finally selected (Fig. 1).

### Attributes of incorporated studies

Each of the incorporated studies constituted randomized, double-blind, placebo-controlled trials predominantly orchestrated at disparate study sites across several countries. A cohort of 10,201 patients diagnosed with eosinophilic asthma were recruited. The demographic included 3938 males, 6238 females, as well as 25 not having disclosed their gender. The participant age spanned from 6 to 82 years. Among the 10,171 treated patients, 6260 were administered anti-IL-5 therapy, while the residual 3911 received a placebo. The female participants were mandated to adopt sufficient contraception from the initial screening until the culmination of the study. Meanwhile, all patients were randomized to receive either anti-IL-5 treatment or placebo as an adjunctive therapy to their standard background regimen, administered via intravenous or subcutaneous injection. This conventional background treatment entailed ICS/OCS/LABA/ LAMA/tiotropium/leukotriene receptor antagonists (LTRAs)/chromone/cromolyn/theophylline/5-lipoxygenase inhibitors. Patients administered ≥1 dose of the study drug were incorporated in the safety analysis. The detailed characteristics of the included studies are encapsulated in Appendix 4.

### Risk of bias in the included studies

**Fig. 2:**
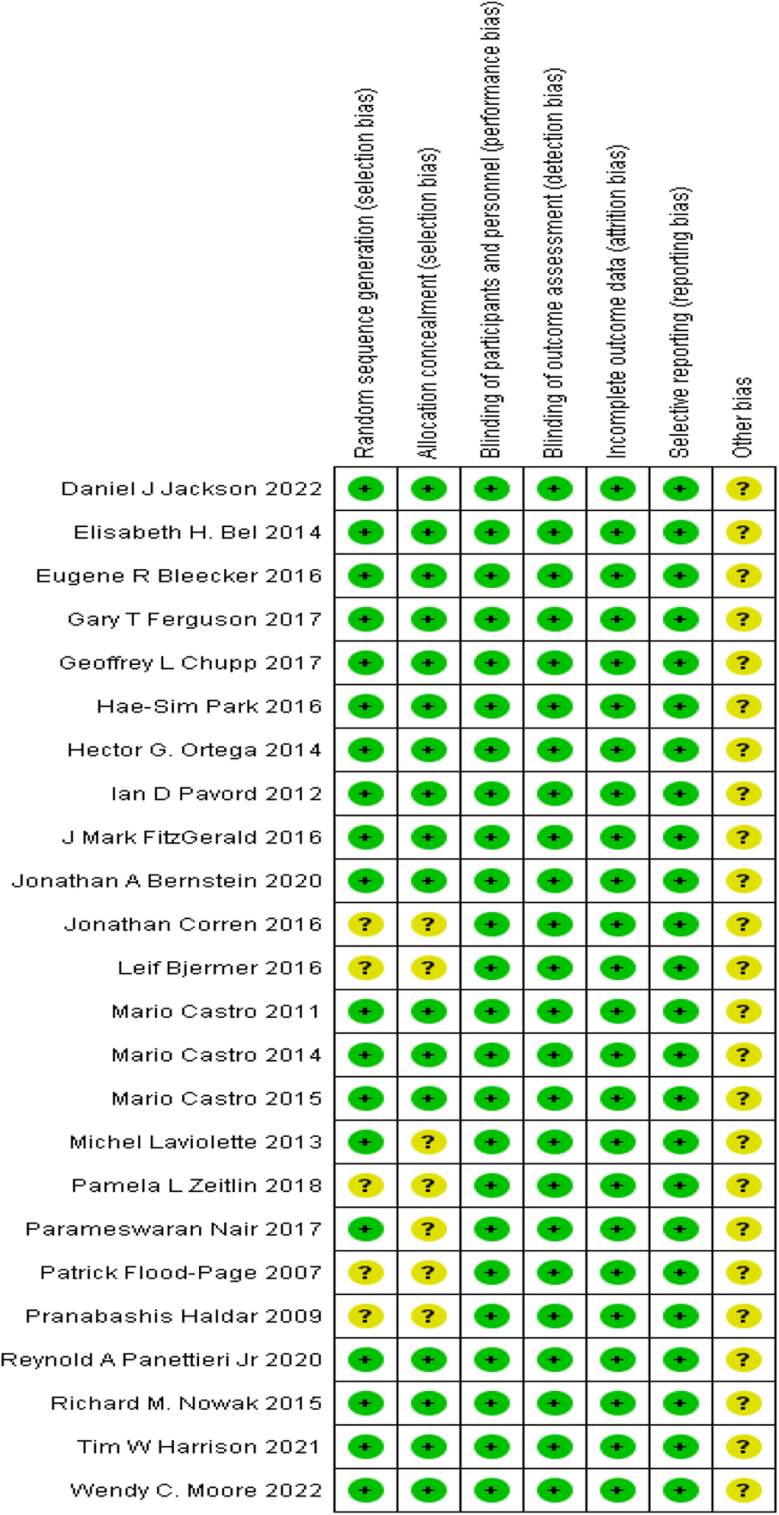
Risk of bias summary for included studies

Among the incorporated studies, 5 [17, 18, 25, 27, 34] failed to stipulate the randomization methodology in their published work, and were hence categorized as being subject to an ambiguous risk of randomization bias. Remaining studies that employed interactive voice response systems, central computer-generated permuted-block design, or sequential randomization code assignment were considered to harbor a low risk of bias. Meanwhile, 7 studies [17, 18, 25, 27, 34, 37, 38] did not articulate allocation concealment and were consequently categorized as bearing an indeterminate risk of assignment bias, while the remaining studies provided allocation concealment. Considering the double-blind design of all studies, the investigators and participants were deemed adequately blinded, indicating a low risk of performance bias across all studies. The outcome of this analysis was not deemed to be affected by the blinding of outcome assessment across all studies, and the detection bias was thus categorized as low risk. Given that each included study demonstrated limited loss to follow-up, provided clear explanations for any loss, and effectively managed missing data, all studies were classified as bearing a low risk of attrition bias. Besides, all predetermined outcomes were reported, so these studies were classified as low risk in terms of reporting bias. Figure 2 presents the assessments for each risk-of-bias item in the included studies.

### Overall AEs

**Fig. 3:**
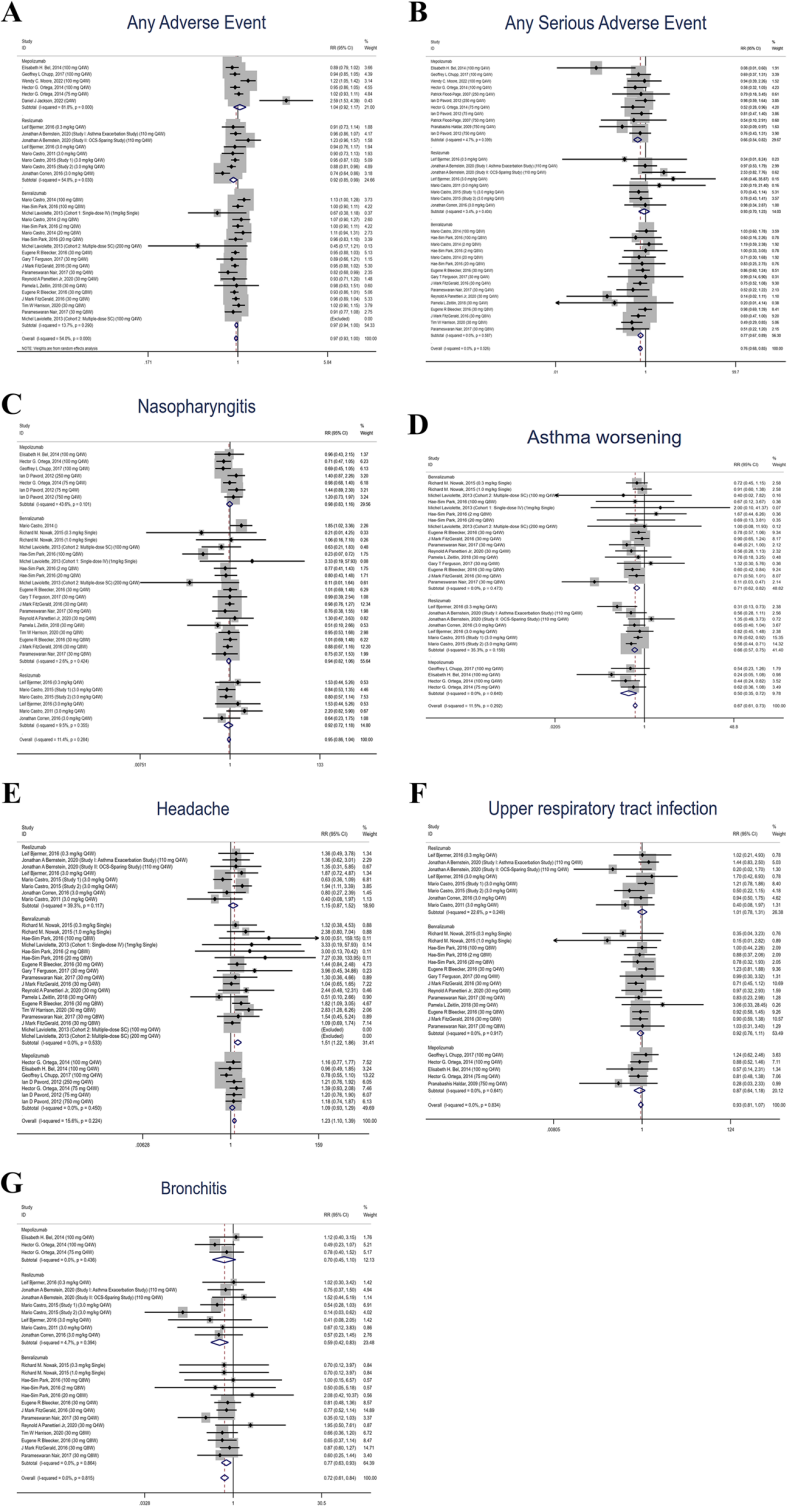
The relationship of overall adverse events between the anti-IL-5 treatment group and the control group for patients with eosinophilic asthma

Overall AEs were documented in 22 studies across 20 published articles: 68.93% (3765/5462) of patients undergoing anti-IL-5 therapy experienced AEs in comparison to 71.84% (3791/5277) in the placebo cohort. The incidence of general AEs in the anti-IL-5 treatment group was congruent with those reported in the placebo group (RR: 0.97, 95% CI: 0.93–1.00, *P* = 0.000, I^2^ = 54.0%). Though not statistically significant, the probability of AEs manifestation in the mepolizumab group vis-à-vis the placebo group was marginally elevated in contrast to the benralizumab and reslizumab treatment groups. Heterogeneity was observed in mepolizumab and reslizumab treatment cohorts. Meanwhile, sensitivity analysis and meta-regression of dosage, route of administration, frequency of administration, and intervention assignment were conducted for the mepolizumab and reslizumab groups, and the analysis results demonstrated dosage, route of administration, and intervention assignment (*P* = 0.000) as contributing factors to heterogeneity in the reslizumab group. Egger’s test indicated no publication bias (*P* = 0.635) (Fig. 3A and Appendix 5A).

### Serious AEs

Serious adverse events (SAEs) were reported in 23 studies across 21 published articles in this cumulative analysis: 8.22% (495/6025) of patients treated with anti-IL-5 drugs experienced SAEs in comparison to 11.20% (657/5867) treated with a placebo. Overall, the incidence of SAEs in patients from the anti-IL-5 treatment group was significantly lower than that in patients from the placebo group (RR: 0.76, 95% CI: 0.68–0.85, *P* = 0.526, I^2^ = 0.0%). This disparity was exclusively observed in patients administered mepolizumab (RR: 0.66, 95% CI: 0.54–0.82, *P* = 0.399, I^2^ = 4.7%) and benralizumab (RR: 0.77, 95% CI: 0.67–0.89, *P* = 0.567, I^2^ = 0.0%). Analysis of publication bias displayed a relatively symmetrical funnel plot, while Egger’s test showed no publication bias (*P* = 0.305). (Fig. 3B and Appendix 5B).

### Common AEs

Nasopharyngitis, asthma worsening, headache, upper respiratory tract infection, and bronchitis were the 5 most common AEs reported in the anti-IL-5 treatment group (noted in all sub-treatment cohorts).

### Nasopharyngitis

Nasopharyngitis was cited in 20 studies from 19 published articles: Patients (13.55%, 729/5380) in the anti-IL-5 treatment group had a similar likelihood of suffering nasopharyngitis compared to those (15.45%, 734/475) in the placebo group (RR: 0.95, 95%CI: 0.86–1.04, *P* = 0.284, I2 = 11.4%). The likelihood of nasopharyngitis manifestation was also similar across sub-treatment groups. Besides, no evident publication bias was observed per Egger’s test (*P* = 0.649). (Fig. 3C and Appendix 5C).

### Asthma worsening

Asthma worsening was documented in 18 studies sourced from 16 published articles: The incidence of this AE was significantly lower in patients (13.31%, 583/4379) receiving anti-IL-5 treatment compared to those (19.99%, 822/4113) treated with a placebo (RR: 0.67, 95%CI: 0.61–0.73, *P* = 0.292, I^2^ = 11.5%). This trend was consistent in all sub-treatment groups. Meanwhile, Egger’s test signified no publication bias (*P* = 0.389). (Fig. 3D and Appendix 5D).

### Headache

The incidence of headache was reported in 21 studies from 19 published articles: Headache occurred in 10.55% (561/5320) of patients undergoing anti-IL-5 treatment and 8.99% (437/4860) placebo patients, 23% higher in the anti-IL-5 treatment group compared to the placebo group (RR: 1.23, 95%CI: 1.10–1.39, *P* = 0.224, I^2^ = 15.6%), presenting the highest prevalence in the benralizumab group (RR: 1.51, 95%CI: 1.22–1.86, *P* = 0.533, I^2^ = 0.0%) among all sub-treatment groups. A skewed funnel plot was observed, and publication bias was statistically significant per Egger’s test (*P* = 0.042). (Fig. 3E and Appendix 5E).

### Upper respiratory tract infection

Upper respiratory tract infection (URTI) was reported in 19 studies from 17 published articles: Patients (8.57%, 381/4444) in the anti-IL-5 treatment group had similar occurrence of upper respiratory infection to those (9.01%, 377/4183) in the placebo group (RR: 0.93, 95%CI: 0.81–1.07, *P* = 0.834, I^2^ = 0.0%), and of 3 sub-treatment groups, patients in the mepolizumab group had relatively less occurrence of upper respiratory infection. Egger’s test signified no publication bias (*P* = 0.051). (Fig. 3F and Appendix 5F).

### Bronchitis

Bronchitis was reported in 16 studies from 14 published articles: Fewer patients (5.98%, 264/4412) in the anti-IL-5 treatment group developed bronchitis in contrast with those (8.64%, 341/3945) in the placebo group (RR: 0.72, 95%CI: 0.61–0.84, *P* = 0.815, I^2^ = 0.0%), and it seemed less possible for patients administered with reslizumab to have bronchitis, relatively speaking of the other 2 sub-treatment groups (RR: 0.59, 95%CI: 0.42–0.83, *P* = 0.394, I^2^ = 4.7%). Egger’s test signified no publication bias (*P* = 0.635). (Fig. 3G and Appendix 5G).

### AEs supplementary

Other adverse events occurred in no less than 2 sub-treatment groups were summarized in Appendix 6. Events that satisfied with the criteria of event incidence in anti-IL5 treatment group ≥2 times incidence in placebo group and event number ≥ 3 included: anxiety, asthenia, elevated blood creatinine, constipation, heightened thermal perception, hyperhidrosis, influenza-mimicking sickness, large intestinal polyps, muscle spasms, presyncope, procedural pain, perennial rhinitis, stomatitis, exclusively in the benralizumab cohort; unspecified local site reactions singularly in the mepolizumab cohort; malignant neoplasms, pharyngolaryngeal pain solely within the reslizumab cohort.

## Results from examination of adverse events from post-marketing reports (PRR and BCPNN)

### Post-marketing report - attributes

From 01-Jan-2018 to 30-Sep-2022, a total of 10,589,313 adverse event instances were gathered from the FAERS, out of which, 28,478 implicated suspect medicinal agents (mepolizumab, benralizumab, and reslizumab). Of this subset, the suspect agents were primarily prescribed for asthma (73.34%). Predominantly, healthcare professionals reported these cases, with the majority of the affected patients being females (53.96%), approximately doubling the number of male patients (26.12%). SAEs represented 65.89% of the total 28,478 reports, with hospitalization (30.30%) being the most frequent incident among these serious occurrences. (Table 1).

**Table 1:** Demographic profile and characterization of adverse event reports of anti-IL-5 drugs

Category	Number of Cases	Percentage
**Indication**		
Asthma	20,886	73.34%
Unknown Indication	6356	22.32%
Other Indication	1236	4.34%
**Reporter Type**		
Healthcare Professional	16,582	58.23%
Consumer	11,758	41.29%
Not Specified	138	0.48%
**Gender**		
Male	7438	26.12%
Female	15,366	53.96%
Not Specified	5674	19.92%
**Age**		
0–1 Month	8	0.03%
2 Months-2 Years	5	0.02%
3–11 Years	66	0.23%
12–17 Years	154	0.54%
18–64 Years	7387	25.94%
65–85 Years	4589	16.11%
More than 85 Years	211	0.74%
Not Specified	16,058	56.39%
**Seriousness Criteria**		
Died	1555	5.46%
Life Threatening	366	1.29%
Hospitalised	8628	30.30%
Disabled	228	0.80%
Congenital Anomaly	70	0.25%
Required Intervention	12	0.04%

### Post-marketing report - signal detection results

Among the top 100 AEs overall associated with anti-IL-5 drugs, 75 suspicious observations emerged, implicating 16 SOCs. (Table 2) The comprehensive safety profile of anti-IL-5 medications, distilled from these 75 suspect observations, was chiefly delineated as a constellation of manifestations associated with the progression of the indicated disease, uncontrolled underlying conditions, and incomplete therapeutic effect, which included clinical signs, symptoms, diminished quality of life, anomalous lab results, etc. Further events were tied to the pharmacological properties and mechanisms of action of the products, such as infections, localized and systemic allergic reactions, etc., also erroneous dosage administration, and exposure to the product arising from inappropriate usage and product issues. These overarching safety characteristics of anti-IL-5 medications, derived from signal detection, displayed general congruity with mepolizumab and benralizumab’s signal detection results, while the reslizumab results did not conspicuously depict events related to infections, product issues, and inappropriate usage (Appendices 7, 8 & 9). As asthma is the overwhelming indication for these anti-IL5 drugs, we assumed the suspect observations could represent the safety concerns indicated for asthma as well.

**Table 2:** Results of signal detection for adverse events of anti-IL-5 drugs

SOC/PT	Event (A)	PRR	X^2^	E (IC)-2SD
**Blood And Lymphatic System Disorders**				
Eosinophilia^a^	328	20.67	5819.59	3.84
**Gastrointestinal Disorders**				
Gastrooesophageal Reflux Disease	350	5.29	1204.78	2.01
**General Disorders And Administration Site Conditions**				
Adverse Drug Reaction	298	3.33	482.35	1.33
Chest Discomfort^a^	1271	15.05	16,068.74	3.65
Chest Pain	502	3.68	973.47	1.56
Chills	341	3.56	622.70	1.45
Condition Aggravated	1091	3.57	2024.68	1.62
Fatigue	1491	2.06	832.45	0.86
Ill-Defined Disorder	350	9.56	2617.90	2.83
Illness	539	2.89	665.60	1.23
Injection Site Pain	527	2.43	445.14	0.98
Malaise	1351	4.30	3416.87	1.90
Oedema Peripheral	279	3.77	564.38	1.49
Pyrexia	1040	3.51	1873.04	1.59
Secretion Discharge^a^	250	21.57	4635.53	3.81
Therapeutic Product Effect Incomplete^a^	3121	23.66	63,994.22	4.34
**Immune System Disorders**				
Anaphylactic Reaction	249	5.41	884.83	1.97
Hypersensitivity	562	3.07	781.31	1.32
**Infections And Infestations**				
Bronchitis	414	6.74	1990.83	2.38
Covid-19	499	2.13	298.28	0.78
Herpes Zoster	288	5.28	988.19	1.97
Infection	501	3.76	1008.76	1.59
Influenza	679	6.74	3272.32	2.46
Lower Respiratory Tract Infection^a^	643	14.77	7951.78	3.53
Nasopharyngitis	947	5.86	3774.96	2.30
Pneumonia	2560	8.71	17,260.09	2.95
Respiratory Tract Infection^a^	375	16.48	5223.38	3.58
Sinusitis	610	6.92	3040.83	2.48
Upper Respiratory Tract Infection	304	8.04	1837.83	2.56
**Injury, Poisoning And Procedural Complications**				
Accidental Exposure To Product	453	4.42	1186.84	1.80
Exposure Via Skin Contact^a^	490	120.58	43,858.25	5.93
Inappropriate Schedule Of Product Administration	776	2.87	946.59	1.27
Product Dose Omission Issue	2199	3.92	4828.04	1.81
Underdose	274	4.14	647.65	1.62
Wrong Technique In Device Usage Process	542	9.18	3861.21	2.85
**Investigations**				
Blood Pressure Increased	446	3.14	646.44	1.32
Blood Test Abnormal^a^	259	18.36	4054.03	3.62
Eosinophil Count Increased^a^	560	78.66	35,435.09	5.56
Full Blood Count Abnormal^a^	1922	60.09	96,236.90	5.49
Heart Rate Increased	282	3.41	478.38	1.35
Oxygen Saturation Decreased	279	5.05	896.95	1.90
Weight Increased	410	2.12	243.80	0.75
**Musculoskeletal And Connective Tissue Disorders**				
Arthralgia	791	2.09	451.03	0.81
Back Pain	696	3.47	1218.45	1.52
Myalgia	420	3.07	584.62	1.28
Pain In Extremity	561	2.24	384.08	0.87
**Nervous System Disorders**				
Headache	1582	2.90	1991.73	1.35
**Product Issues**				
Product Complaint^a^	515	12.17	5120.31	3.23
**Psychiatric Disorders**				
Sleep Disorder	247	3.89	526.57	1.51
Sleep Disorder Due To A General Medical Condition^a^	1766	189.72	219,426.83	6.69
**Respiratory, Thoracic And Mediastinal Disorders**				
Asthma^a^	8071	95.40	601,908.84	6.15
Asthmatic Crisis^a^	486	172.80	56,633.75	6.23
Bronchiectasis^a^	313	54.01	14,218.65	4.96
Chronic Obstructive Pulmonary Disease^a^	554	13.09	5985.27	3.34
Cough^a^	3162	12.62	33,004.38	3.48
Dysphonia	317	6.19	1357.85	2.21
Dyspnoea^a^	6395	13.22	70,989.84	3.58
Dyspnoea Exertional^a^	724	19.89	12,344.85	3.95
Lung Disorder	436	9.95	3421.92	2.93
Nasal Congestion^a^	667	13.45	7429.47	3.41
Nasal Polyps^a^	355	139.31	35,440.36	5.91
Obstructive Airways Disorder^a^	1420	133.11	137,080.89	6.32
Oropharyngeal Pain	510	6.17	2178.26	2.29
Productive Cough^a^	1413	32.38	39,585.24	4.68
Pulmonary Mass^a^	291	19.70	4906.56	3.74
Respiratory Disorder^a^	291	11.76	2779.49	3.07
Rhinorrhoea	463	7.44	2536.10	2.54
Sputum Discoloured^a^	683	77.22	42,544.86	5.60
Upper-Airway Cough Syndrome^a^	285	40.57	9917.96	4.61
Wheezing^a^	4134	90.04	293,468.35	6.04
**Skin And Subcutaneous Tissue Disorders**				
Rash	845	2.03	448.55	0.79
Urticaria	550	3.84	1146.86	1.63
**Social Circumstances**				
Loss Of Personal Independence In Daily Activities^a^	2492	39.39	84,484.01	4.99
**Surgical And Medical Procedures**				
Hospitalisation	927	6.17	3974.32	2.37
**Vascular Disorders**				
Hypertension	555	3.13	804.20	1.35

^a^Indicates strong signals

Among the top 100 AEs each associated with mepolizumab, benralizumab, and reslizumab, 78 suspect observations were identified for mepolizumab, 66 for benralizumab, and 24 for reslizumab, respectively. Considering the diverse application of these drugs, not solely confined to treating asthma but also for eosinophilic granulomatosis with polyangiitis, nasal polyps, chronic obstructive pulmonary disease, along with other undetermined and off-label indications, as well as concurrent medications reported in post-marketing cases, several events could potentially be attributed to complications of underlying diseases. These may include thoracic and respiratory signs and symptoms, diminished quality of life - encompassing sleep disorders and impaired daily activities, abnormal laboratory findings such as decreased oxygen saturation, increased eosinophil count, and aberrant breath sounds. Furthermore, several consequences could be potentially attributable to the routine concomitant administration of corticosteroids, bronchodilators, 5-lipoxygenase inhibitors, and leukotriene receptor antagonists, which included events concerning eye disorders such as cataracts; endocrine alterations such as hyperglycemia, adrenal suppression, and weight gain; various infections encompassing opportunistic infections; gastrointestinal discomforts such as gastroesophageal reflux and nausea; cardiac issues such as tachycardia and hypertension; musculoskeletal pain; as well as general symptoms comprising weakness, malaise, fatigue, headaches, dysphonia, and sleep disorders. Meanwhile, cutaneous reactions including urticaria, pruritus, and rashes could potentially be linked to listed hypersensitivity reactions to anti-IL-5 medications. Though bearing alternative interpretations, these observations remain crucial to maintain continuous monitoring to discern potential causal relationships with anti-IL-5 drugs, and attention should be heightened towards new and significant signals identified during this signal detection process. Newly-detected potential signals of incomplete therapeutic product effects from mepolizumab might contribute to the worsening of underlying diseases, resulting in considerable clinical signs, symptoms, complications, and abnormal lab results. Improper product exposure, device leakage, and incorrect dose administration might result from issues with device usage techniques and product issues detected from both mepolizumab and benralizumab, while adverse effects like chronic sinusitis, sinusitis, and nasopharyngitis detected from mepolizumab might induce symptoms such as headaches, nasal congestion, rhinorrhea, and oropharyngeal pain, all falling under the umbrella of URTI. Besides, pulmonary masses identified in mepolizumab patients might be accompanied with adverse respiratory implications, necessitating further investigations to determine the nature of the mass (Appendices 7, 8 & 9).

## Discussion

He [50], Wang [51] and Akenroye [52] conducted a meta-analysis of anti-IL-5 therapies, focusing on efficacy and safety, however, the safety analysis lacked details, and AEs were not clearly specified. This underscores the necessity for a comprehensive understanding of the safety profiles of these medications, encompassing both shared and unique safety data for each drug, and the identification of potential safety concerns emerging from post-marketing experiences.

Analysis of AEs and SAEs, revealed no dose-related trends within sub-treatment groups. Consistent with most studies, AE rates in patients receiving anti-IL-5 therapy paralleled those in placebo groups. Significantly, patients treated with anti-IL-5 drugs, particularly with mepolizumab and benralizumab, showed a marked decrease in SAE risk. Overall, anti-IL-5 medications demonstrate strong tolerability in treating eosinophilic asthma.

IL-5 involvement in pathophysiology of migraine remains unclear [53, 54]. The acquired clinical data demonstrated a markedly elevated incidence of cephalgia in the anti-IL-5 treatment cohort compared to the placebo group, presenting particularly prominent disparity in the benralizumab subgroup. Post-marketing reports also exhibited potential indications of cephalgia for all anti-IL5 pharmaceuticals, rendering it a confirmed adverse event within mepolizumab and benralizumab product descriptions. In the absence of definitive pharmacological research validating whether anti-IL-5 drugs engender headache, current data and observed reports suggested a potential correlation. Further exploration of cephalgia occurrences reported from reslizumab is thus warranted.

A comprehensive analysis of clinical data elucidated that the incidence rates of URTI, nasopharyngitis, and sinusitis were commensurate or less in the anti-IL-5 treated group relative to the placebo cohort, subject to comparable relative risks across each therapeutic subgroup. Notwithstanding, suspicious observations of these events were identified within post-marketing records of mepolizumab and benralizumab. Meanwhile, acute sinusitis demonstrated a diminished risk in both benralizumab and reslizumab groups, and was not noted among the foremost 100 AEs for any anti-IL-5 drugs within post-marketing documentation. In contrast, clinical data suggested an elevated incidence of pharyngitis within the benralizumab subgroup as well as a decrease in the reslizumab subgroup. Despite the absence of clinical data linking mepolizumab to pharyngitis, this condition was designated as an adverse event in product information for both mepolizumab and benralizumab. Furthermore, possibly owing to the limited range of AEs screened for signal detection, pharyngitis was not observed in the top 100 AEs for anti-IL-5 drugs. Chronic sinusitis clinical data were not available in the published literature, but were manifested as a strong suspicious observation solely within mepolizumab’s post-marketing reports, while rhinitis incidence appeared commensurate between the benralizumab and placebo cohorts, but was considerably higher within the mepolizumab subgroup. The limited published data available for mepolizumab were insufficient to establish a possibility between mepolizumab and rhinitis. Additionally, rhinitis was absent in any post-marketing documentation of anti-IL-5 pharmaceuticals. Comparative analysis suggested the reduced likelihood of reslizumab of inciting URTIs based on clinical and post-marketing data. Nonetheless, animal studies indicated that mice deficient in IL-5 or IL-5 receptor genes exhibited numerous developmental and functional B cell deficits, which potentially led to URTIs associated with encapsulated bacteria [55–57]. Given the mode of action of anti-IL-5 pharmaceuticals, potential effects on the onset of URTIs could not be definitively excluded. Other concurrent asthma therapies, such as corticosteroids, could exert an immunosuppressive influence, thereby contributing to infection rates. To this end, vigilance should be considered essential in monitoring URTIs during anti-IL-5 treatment, and causal relationships must be meticulously evaluated.

Besides, analysis of clinical trial data demonstrated a considerable elevation in relative risk for injection/infusion site reactions in both mepolizumab and benralizumab cohorts, designating these events as identified risks within both product labels. Reports of these reactions also emerged from post-marketing surveillance of all anti-IL-5 drugs. Although injection/infusion site reactions were not listed as AEs during reslizumab labeling, and their incidence rate did not significantly exceed that of the placebo group, the dosage form and administration route of the drug warrant routine monitoring and thorough assessment of such reactions.

Cytokines such as IL-5, implicated in asthma pathogenesis, are not recognized as potent pyrogens and are thus unlikely to precipitate fever in asthma patients [58]. The role of anti-IL-5 drugs in the genesis of pyrexia remains ambiguous. Liu, W., et al. [59] postulated a potential correlation with drug pharmacokinetics. The present clinical data indicated a higher occurrence of pyrexia in the benralizumab cohort compared to the placebo group, also a reduced likelihood in the mepolizumab group. Given the limited number of published studies of mepolizumab, the current clinical data were inadequate to establish a causal link between mepolizumab and pyrexia [22]. However, post-marketing reports and product inserts for mepolizumab and benralizumab revealed indications of pyrexia, suggesting a potential association between these anti-IL-5 drugs and pyrexia.

In clinical trials, the relative risk of hypersensitivity events was marginally increased within anti-IL-5 treatment groups, but no significant disparity was observed when compared to placebo groups. Despite the lack of statistical significance within the clinical data, post-marketing surveillance detected hypersensitivity signals across all anti-IL-5 drugs, which were subsequently confirmed within each product labeling. Furthermore, the composition of these drugs could potentially elicit hypersensitivity reactions.

Although the efficacy of treating asthmatic patients with anti-IL-5 drugs was proved in considerable clinical studies, some [18, 23, 25, 30] suggested that less clinical benefit was obtained with anti-IL-5 treatments. In the present study, the clinical data did not suggest a lack of efficacy. On the contrary, the clinical analysis indicated that asthma development, including its exacerbation, was less likely in the anti-IL-5 treatment group compared to the placebo group. However, the post-marketing analysis inferred potential AEs related to disease progression, including symptomology, laboratory test results, diminished quality of life, daily activity impairment, etc., further highlighting a potential signal of incomplete therapeutic effect observed across all anti-IL-5 drugs, especially a strong signal noticed from mepolizumab reports. We speculated that mepolizumab might be less effective than reslizumab and benralizumab due to deficient eosinophil suppression [60–62]. Product inserts for mepolizumab, benralizumab, and reslizumab cautioned potential occurrence of asthma-related adverse symptoms, exacerbations, or uncontrollable conditions post-treatment initiation [41–43]. It was hereby presumed that this discrepancy could result from effective treatment management during clinical trials, while real-world clinical assessments and medication applications might present increased complexity. The post-marketing analysis raises queries about the adequacy of asthma treatment in real-world clinical practice. In light of numerous post-marketing reports, the potential signal of an incomplete therapeutic effect warrants further evaluation.

Notably, potential signals concerning improper device usage and product issues were solely observed in the post-marketing reports for mepolizumab and benralizumab. Herein, it was hypothesized that these AEs might transpire due to the pharmaceutical forms of both drugs. All anti-IL-5 drugs were administered in solution form, with subcutaneous or intravenous injection as the principal routes, thereby possibly enhancing the risk of product exposure. Immediate interventions should be undertaken to address safety concerns caused by inappropriate usage and product issues, such as unexpected product exposure, incorrect dose administration, and accidental injuries.

Sum up, both clinical meta-analysis and post-marketing signal detection have identified potential risks for anti-IL-5 medications, including headaches, reactions at injection or infusion sites, fever, and hypersensitivity. Additionally, respiratory infection-related events, asthma exacerbations, misuse, and product quality concerns were primarily reported in post-marketing spontaneous reports. This discrepancy may be attributed to limited sample sizes and stringent controls in clinical trials. Confounding factors, including partial data from spontaneous reports, complications arising from concurrent medications and pre-existing conditions, and inconsistent clinical practices observed in post-marketing scenarios, might skew the results obtained from post-marketing signal detection. Nonetheless, it is important to acknowledge that these influences, while significant, cannot be completely discounted.

The present integration of clinical and post-marketing data offers a comprehensive safety profile of anti-IL-5 drugs. Nevertheless, the study is still subject to certain limitations. Initially, only a fraction of AEs was published and extractable from literature. Additionally, the small number of studies included made it challenging to assess publication bias and identify heterogeneity factors. Finally, quantitative analysis formed part of the signal detection process to identify suspect observations, and case reviews and alternative explanations should be considered when evaluating causal relationships.

## Conclusion

Upon the evaluation of clinical and post-marketing safety data, the safety profile of each drug generally aligned with the relevant product insert. The comprehensive safety profile of anti-IL-5 drugs predominantly embodied manifestations associated with disease progression and incomplete therapeutic effect. AEs including infections, hypersensitivity, headache, pyrexia, and injection/infusion site reactions, were related to product pharmacological properties and the mode of action, while incorrect dose administration and product exposure were tied to inappropriate usage and product issue. Mepolizumab and benralizumab shared overall safety characteristics, whereas events of infections, pyrexia, product issues, and inappropriate use were absent in reslizumab. Incorrect device usage, product issues, and URTIs were suspect signals for mepolizumab and benralizumab. Meanwhile, an incomplete therapeutic effect surfaced as a new suspect signal for all anti-IL-5 drugs. The potential rise in the incidence of incomplete therapeutic effects could adversely impact the benefit-risk profile of anti-IL-5 drugs, prompting a re-evaluation of their clinical value. Overall, the suspect observation of an incomplete therapeutic effect warrants ongoing investigation and meticulous assessment.

### Supplementary Information

**Additional file 1.**

## Data Availability

Wen Li, Shi-Chao Tang and Lei Jin had full access to all the data in the study and take responsibility for the integrity of the data and the accuracy of the data analysis. Data will be provided under request to the first authors.

## Abbreviations

AEs: Adverse events
SAEs: Serious adverse events
SOCs: System organ classes
BEC: Blood eosinophil count
ICS: Inhaled corticosteroids
OCS: Oral corticosteroids
LABA: Long-acting β2 agonists
LAMA: Long-acting muscarinic receptor antagonist
IL-5: Interleukin-5
RCT: Randomized controlled trials
RRs: Risk ratios
CIs: confidence intervals
FAERS: FDA Adverse Event Reporting System
PTs: Preferred Terms
PRR: Proportional reporting rate
BCPNN: Bayesian Confidence Propagation Neural Network
LTRAs: Leukotriene receptor antagonists
URTI: Upper respiratory tract infection
