# Supplementary material for: Adverse events of anti-IL-5 drugs in patients with eosinophilic asthma: a meta-analysis of randomized controlled trials and real-world evidence-based assessments
Source: BMC Pulm Med. 2024 Feb 3;24:70. doi: 10.1186/s12890-024-02885-2 (PMC10837872; doi:10.1186/s12890-024-02885-2)
Supplement: Supplementary file 1 — Additional file 1. [file 12890_2024_2885_MOESM1_ESM.docx]

# **Supplementary Appendices**

## **Appendix 1**

| **PubMed Search Strategy** | |
| --- | --- |
| **Search items** | **Search Details** |
| #1 | ((("mepolizumab" [Supplementary Concept]) OR (((((mepolizumab[Title/Abstract]) OR (Bosatria[Title/Abstract])) OR (SB-240563[Title/Abstract])) OR (SB240563[Title/Abstract])) OR (Nucala[Title/Abstract]))) AND (("Asthma"[Mesh]) OR ((((Asthma[Title/Abstract]) OR (Asthmas[Title/Abstract])) OR (Bronchial Asthma[Title/Abstract])) OR (Asthma, Bronchial[Title/Abstract])))) AND (("1900"[Date - Publication] : "2022/09/30"[Date - Publication])) |
| #2 | ((("benralizumab" [Supplementary Concept]) OR (((((benralizumab[Title/Abstract]) OR (MEDI-563[Title/Abstract])) OR (MEDI 563[Title/Abstract])) OR (Fasenra[Title/Abstract])) OR (BIW-8405[Title/Abstract]))) AND (("Asthma"[Mesh]) OR ((((Asthma[Title/Abstract]) OR (Asthmas[Title/Abstract])) OR (Bronchial Asthma[Title/Abstract])) OR (Asthma, Bronchial[Title/Abstract])))) AND (("1900"[Date - Publication] : "2022/09/30"[Date - Publication])) |
| #3 | ((("reslizumab" [Supplementary Concept]) OR ((((((((((reslizumab[Title/Abstract]) OR (SCH-55700[Title/Abstract])) OR (SCH55700[Title/Abstract])) OR (SCH 55700[Title/Abstract])) OR (CEP-38072[Title/Abstract])) OR (CEP38072[Title/Abstract])) OR (Cinqair[Title/Abstract])) OR (DCP-835[Title/Abstract])) OR (DCP835[Title/Abstract])) OR (DCP 835[Title/Abstract]))) AND (("Asthma"[Mesh]) OR ((((Asthma[Title/Abstract]) OR (Asthmas[Title/Abstract])) OR (Bronchial Asthma[Title/Abstract])) OR (Asthma, Bronchial[Title/Abstract])))) AND (("1900"[Date - Publication] : "2022/09/30"[Date - Publication])) |

## **Appendix 2**

| **2x2 Contingency Table** | | | |
| --- | --- | --- | --- |
|  | Target event | Other events | Total |
| Suspect medicinal product | A | B | A+B |
| Other medicinal products | C | D | C+D |
| Total | A+C | B+D | N=A+B+C+D |

## **Appendix 3**

| **Formula for signal detection and criteria for signal generation** | | |
| --- | --- | --- |
| **Method** | **Formula** | **Criteria for signal generation** |
| **PRR** | 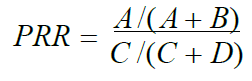  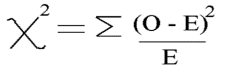 | ① PRR ≥ 2；and  ② X^2^ ≥ 4；and  ③ A≥3 |
| **BCPNN** | \| E (IC) = Log_2_ \| (A + γ_11_)(N + α)(N + β) \| \| --- \| --- \| \| (N + γ)( N_x_ + α_1_)( N_y_ + β_1_) \|  \| Var (IC) = \| 1 \| [ \| N - A + γ - γ_11_ \| + \| N - N_x_ + α - α_1_ \| + \| N - N_y_ + β - β_1_ \| ] \| \| --- \| --- \| --- \| --- \| --- \| --- \| --- \| --- \| --- \| \| (Log2)^2^ \| (A + γ_11_)( 1 + N + γ) \| (N_x_ + α_1_)( 1 + N + α) \| (N_y_ + β_1_)( 1 + N + β) \|  \| γ = γ_11_ \| (N + α)(N + β) \| \| --- \| --- \| \| ( N_x_ + α_1_)( N_y_ + β_1_) \|   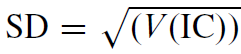 | ① A≥3; and  ② E (IC) - 2SD > 0 |
| Note: γ_11_= 1, α_1_ = β_1_ = 1, α = β = 2, N_x_ = A+B, N_y_ = A+C | | |

## **Appendix 4**

| **Characteristics of Included Studies** | | | | | | | | | |
| --- | --- | --- | --- | --- | --- | --- | --- | --- | --- |
| **Literature (Author, Year)** | **Study Design** | **Subjects** | | | **Intervention** | | | | **Follow-up (Ws)** |
|  |  | **Male / Randomised** | **Age (Y)** | **Type of Asthma** | **Background Treatment** | **Investigational Drug** | **Control** | **Routine** |  |
| Elisabeth H. Bel, 2014 | mRCT, DB, PC | 61/135 | ≥12 | Severe Eosinophilic Asthma | Optimization phase: OCS dose was reduced weekly until an exacerbation; Induction phase: optimized  dose of OCS; Reduction  phase: OCS dose was reduced by 1.25 to 10 mg per day every  4 weeks; Maintenance phase: no adjustment of OCS dose | Mepolizumab 100mg (n=69) Q4W on W0,4,8,12,16,20 | placebo (n=66) | SC | 32 |
| Jonathan A Bernstein, 2020 | mRCT, DB, PC | 252/645 (including 4 with unknown gender) | ≥12 | Severe Asthma | OCS | Reslizumab 110 mg (n=325) Q4W | placebo (n=320) | SC | 64 and 32 |
| Jonathan A Bernstein, 2020 (Study I: Asthma Exacerbation Study) | mRCT, DB, PC | 192/468 (including 4 with unknown gender) | ≥12 | Severe Uncontrolled Asthma | OCS (prednisone 10 mg or less daily, or equivalent) | 13 doses of Reslizumab 110 mg (n=237) Q4W from W0 to W48 | placebo (n=231) | SC | 64 |
| Jonathan A Bernstein, 2020 (Study II: OCS-Sparing Study) | mRCT, DB, PC | 60/177 | ≥12 | Severe Oral  Corticosteroid-dependent Asthma | Optimisation period: OCS dose was reduced at 1-week intervals for up to 10  weeks until a worsening of asthma; Run-in and Induction period: minimally  effective OCS dose (between ≥5 mg and ≤ 40 mg of prednisone daily); Reduction  period: OCS dose was tapered per protocol | 6 doses of Reslizumab 110 mg (n=88) Q4W from W4 to W20 | placebo (n=89) | SC | 32 |
| Leif Bjermer, 2016 | mRCT, DB, PC | 126/315 | 12-75 | Inadequately Controlled  Eosinophilic Asthma | Medium-dose ICS (fluticasone propionate  ≥ 440 mg/d or equivalent) or long-acting bronchodilators or  leukotriene inhibitors or cromolyn | 4 doses of Reslizumab 0.3 mg/kg (n=103) or 3.0 mg/kg (n=103) Q4W from W4 to W16 | placebo (n=105) | IV | 29 |
| Eugene R Bleecker, 2016 | mRCT, DB, PC | 408/1205 (including 1 with unknown gender) | 12-75 | Severe Uncontrolled Asthma | Asthma controller treatments (ICS, LABA, LAMA, tiotropium, LTRAs, chromone, theophylline, OCS) at a stable dosage | Benralizumab 30 mg Q4W (n=403) from W0 to W44 or Benralizumab 30 mg Q8W (n=394) (Q4W on first three doses) on W0, 4, 8, 16, 24, 32, 40 | placebo (n=407) | SC | 56 |
| Mario Castro, 2011 | mRCT, DB, PC | 43/106 | 18-75 | Poorly Controlled Eosinophilic Asthma | ICS (≥440 μg  twice per day of fluticasone or equivalent), LABA, leukotriene antagonists, cromolyn sodium at stable doses | Reslizumab 3.0 mg/kg (n=53) Q4W from W0 to W12 | placebo (n=53) | IV | 24 |
| Mario Castro, 2014 | mRCT, DB, PC | 189/609 (including 3 with unknown gender) | 18-75 | Uncontrolled Asthma | ICS, LABA | Benralizumab 2 mg (n=81), 20 mg (n=81), 100 mg n=223) Q4W on first three dose, then Q8W on  W16, 24, 32, 40 | placebo (n=221) | SC | 66 |
| Mario Castro, 2015 | mRCT, DB, PC | 356/953 | 12-75 | Inadequately Controlled  Eosinophilic Asthma | OCS (a maximum dose of 10 milligrams of prednisone daily or equivalent), SABA, LABA | 13 doses of Reslizumab 3.0 mg/kg (n=477) Q4W to W48 | placebo (n=475) | IV | 66 |
| Mario Castro, 2015 (Study 1) | mRCT, DB, PC | 186/489 | 12-75 | Inadequately Controlled  Eosinophilic Asthma | OCS (a maximum dose of 10 milligrams of prednisone daily or equivalent), SABA, LABA | 13 doses of Reslizumab 3.0 mg/kg (n=245) Q4W to W48 | placebo (n=243) | IV | 66 |
| Mario Castro, 2015 (Study 2) | mRCT, DB, PC | 170/464 | 12-75 | Inadequately Controlled  Eosinophilic Asthma | OCS (a maximum dose of 10 milligrams of prednisone daily or equivalent), SABA, LABA | 13 doses of Reslizumab 3.0 mg/kg (n=232) Q4W to W48 | placebo (n=232) | IV | 66 |
| Geoffrey L Chupp, 2017 | mRCT, DB, PC | 226/556 (including 5 with unknown gender) | ≥12 | Severe Eosinophilic Asthma | ICS, OCS, LABA, LRA, LAMA | Mepolizumab 100 mg (n=273) Q4W from W0 to W20 | placebo (n=278) | SC | 32 |
| Jonathan Corren, 2016 | mRCT, DB, PC | 181/496 | 18-65 | Poorly Controlled Asthma | ICS, long-acting  b-agonists (LABAs), leukotriene-receptor antagonists, 5-lipoxygengase  inhibitors, cromolyn | 4 doses of Reslizumab 3.0 mg/kg (n=395) Q4W from W0 to W12 | placebo (n=97) | IV | 28 |
| Gary T Ferguson, 2017 | mRCT, DB, PC | 82/211 | 18-75 | Mild to Moderate Persistent  Asthma | Either 180 μg or 200 μg of budesonide dry powder inhaler twice daily or equivalent | Benralizumab 30 mg (n=106) Q4w on  W0, 4, 8 | placebo (n=105) | SC | 20 |
| J Mark FitzGerald, 2016 | mRCT, DB, PC | 499/1306 | 12-75 | Severe  Uncontrolled Eosinophilic Asthma | ICS, OCS, LABA, tiotropium, LTRAs, chromone, theophylline | Benralizumab 30 mg Q4W (n=438) from W0 to W52 or Benralizumab 30 mg Q8W (n=428) (Q4W on first three doses, then Q8W on  W16,24,32,40,48) | placebo (n=440) | SC | 60 |
| Patrick Flood-Page, 2007 | mRCT, DB, PC | 160/362 | 18-55 | Moderate Persistent Asthma | ICS, β2-Agonist | 3  doses of Mepolizumab 750 mg (n=116)  or Mepolizumab 250 mg (n=120) Q4W on  W0, 4,  8 | placebo (n=126) | IV | 20 |
| Pranabashis Haldar, 2009 | sRCT, DB, PC | 32/61 | ≥18 | Refractory Eosinophilic Asthma | Oral  prednisolone at a dose of  0.5 mg per kilogram per day, with a maximum  dose of 40 mg per day | 12 doses of Mepolizumab 750 mg (n=29) monthly from W0 to W48 | placebo (n=32) | IV | 52 |
| Tim W Harrison, 2020 | mRCT, DB, PC | 257/660 (including 4 with unknown gender) | 18-75 | Severe Eosinophilic Asthma | Regular asthma controller medications allowed | Benralizumab 30 mg (n=427) Q8W (Q4W on first three doses, then Q8W on  W16) | placebo (n=229) | SC | 26 |
| Daniel J Jackson, 2022 | mRCT, DB, PC | 164/290 | 6-17 | Exacerbation-prone  Eosinophilic Asthma | OCS, LABA | Mepolizumab (6–11 years: 40 mg; 12–17 years:  100 mg) (n=146) Q4W from W4 for 52 weeks | placebo (n=144) | SC | 56 |
| Michel Laviolette, 2013 | mRCT, DB, PC | 11/27 | 18-65 | Eosinophilic Asthma | Regular  asthma medications allowed | Benralizumab (n=17) | placebo (n=10) | IV and SC | 12 and 20 |
| Michel Laviolette, 2013 (Cohort 1: Single-dose IV) | mRCT, DB, PC | 5/13 | 18-65 | Eosinophilic Asthma | Regular  asthma medications allowed | Single dose of Benralizumab 1mg/kg IV (n=8) on W0 | placebo (n=5) | IV | 12 |
| Michel Laviolette, 2013 (Cohort 2: Multiple-dose SC) | mRCT, DB, PC | 6/14 | 18-65 | Eosinophilic Asthma | Regular  asthma medications allowed | 3 doses of Benralizumab100 (n=4) or 200mg (n=5) SC monthly on  W0,4,8 | placebo (n=5) | SC | 20 |
| Wendy C. Moore, 2022 | mRCT, DB, PC | 122/295 | ≥12 | Severe Eosinophilic Asthma | Standard of care allowed | Mepolizumab 100 mg (n=144) Q4W from W0 to W48 | placebo (n=151) | SC | 52 |
| Parameswaran Nair, 2017 | mRCT, DB, PC | 85/220 | 18-75 | Severe Eosinophilic Asthma | ICS, LABA and  any other asthma-controller medications aside  from OCS (including leukotriene  modifiers, long-acting muscarinic antagonists,  and theophylline), in an unchanged fashion allowed  throughout the trial | Benralizumab 30 mg Q4W (n=72) from W4 to W24 or Benralizumab 30 mg Q8W (n=73) (Q4W on first  three doses, then Q8W on W16, 24) | placebo (n=75) | SC | 36 |
| Richard M. Nowak, 2015 | mRCT, DB, PC | 33/110 | 18-60 | Inadequately Controlled Asthma | ICS, LABA, LAMA, LTRI | Single dose of Benralizumab 0.3 mg/kg (n=36) or 1 mg/kg (n=36) | placebo (n=38) | IV | 24 |
| Hector G. Ortega, 2014 | mRCT, DB, PC | 247/576 | 12-82 | Severe Eosinophilic Asthma | Regular  antiasthma therapy allowed | Mepolizumab 75 mg IV (n=191) or Mepolizumab  100 mg SC (n=194)  Q4W  from W0 to W28 | placebo (n=191) | IV and SC | 40 |
| Reynold A Panettieri Jr, 2020 | mRCT, DB, PC | 76/233 | 18-75 | Severe Eosinophilic Asthma | ICS, allergen Immunotherapy allowed if on stable | Benralizumab 30 mg (n=118) Q4W on W0, 4, 8 | placebo (n=115) | SC | 16 |
| Hae-Sim Park, 2016 | mRCT, DB, PC | 38/106 (including 3 with unknown gender) | 20-75 | Eosinophilic Asthma | Same dose of ICS/LABA from the start of  the screening period until week 52 | Benralizumab 2 mg (n=26) or Benralizumab 20 mg (n=25) or Benralizumab 100 mg (n=26) Q8W (Q4W on first  three doses, then Q8W on W16, 24, 32, 40) | placebo (n=26) | SC | 68 |
| Ian D Pavord, 2012 | mRCT, DB, PC | 229/621 (including 5 with unknown gender) | 12-74 | Severe Eosinophilic Asthma | Standard of care allowed | 13 doses of Mepolizumab  75 mg (n=153) or Mepolizumab 250  mg (n=152) or Mepolizumab 750 mg (n=156) Q4W | placebo (n=155) | IV | 56 |
| Pamela L Zeitlin, 2018 | RCT, DB, PC | 61/103 | 12-21 | Moderate to Severe  Asthma | ICS, LABA | Benralizumab 30 mg (n=51) Q4W on W0, 4, 8 | placebo (n=52) | SC | 20 |
| Note: mRCT=multicenter randomised controlled trial, sRCT=single randomised controlled trial, DB=double blind, PC=placebo controlled, IV=intravenous injection, SC=subcutaneous injection, Q4W=every 4 weeks for one dose, Q8W=every 8 weeks for one dose, Ws=weeks, Y=years. | | | | | | | | | |

## **Appendix 5**

**Funnel plot analysis of potential publication bias**

A.

**
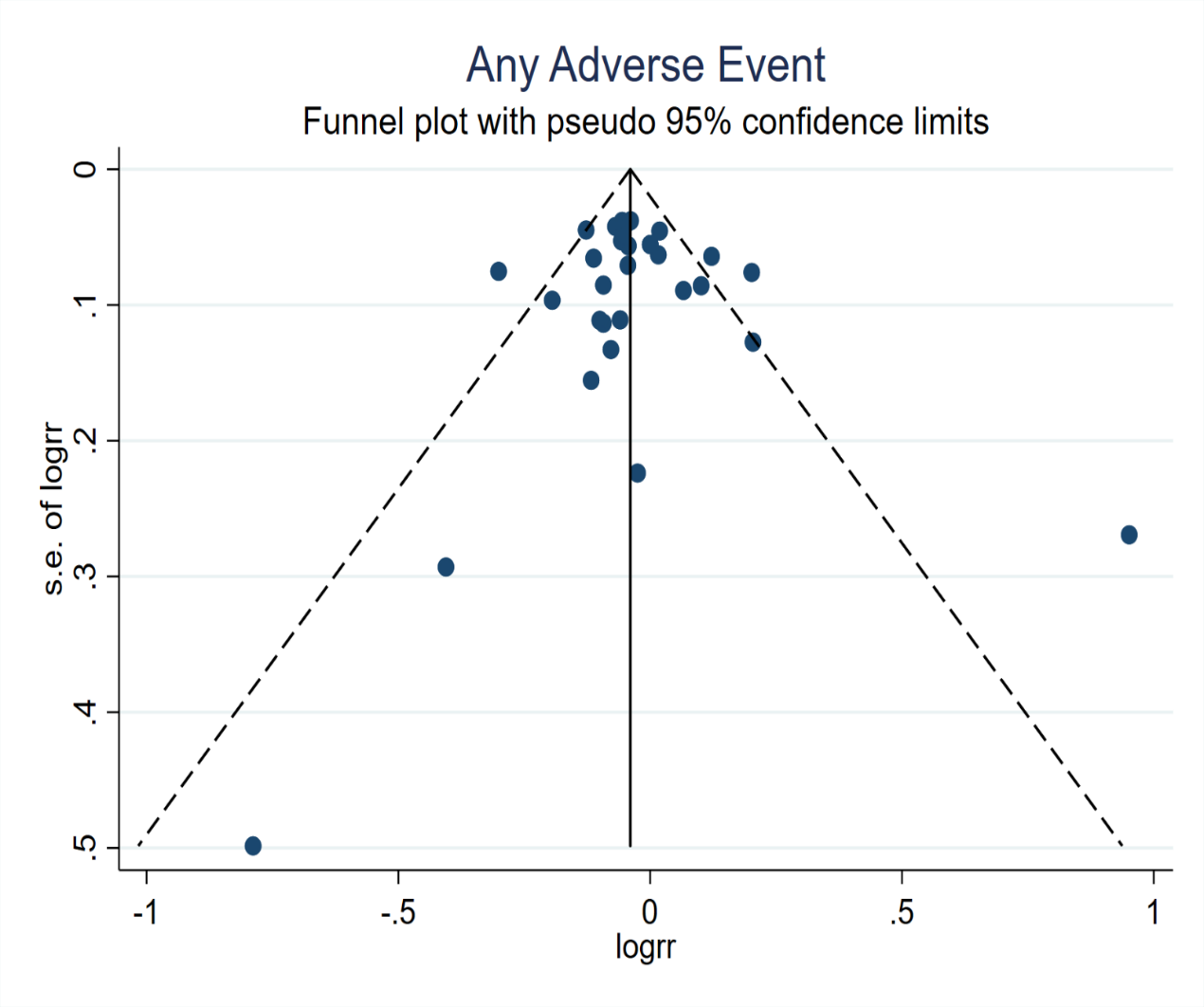
**

B.

**
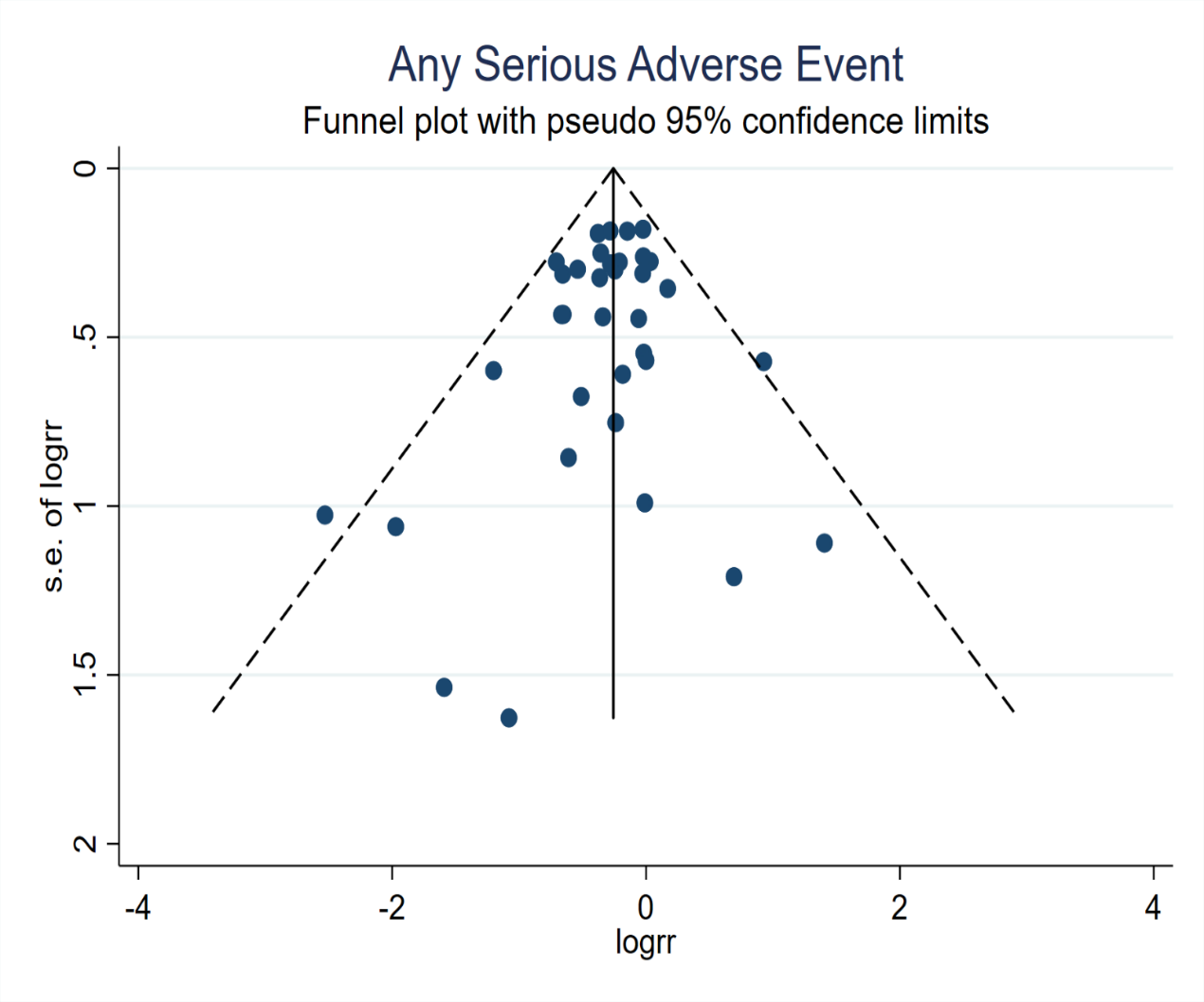
**

C.

**
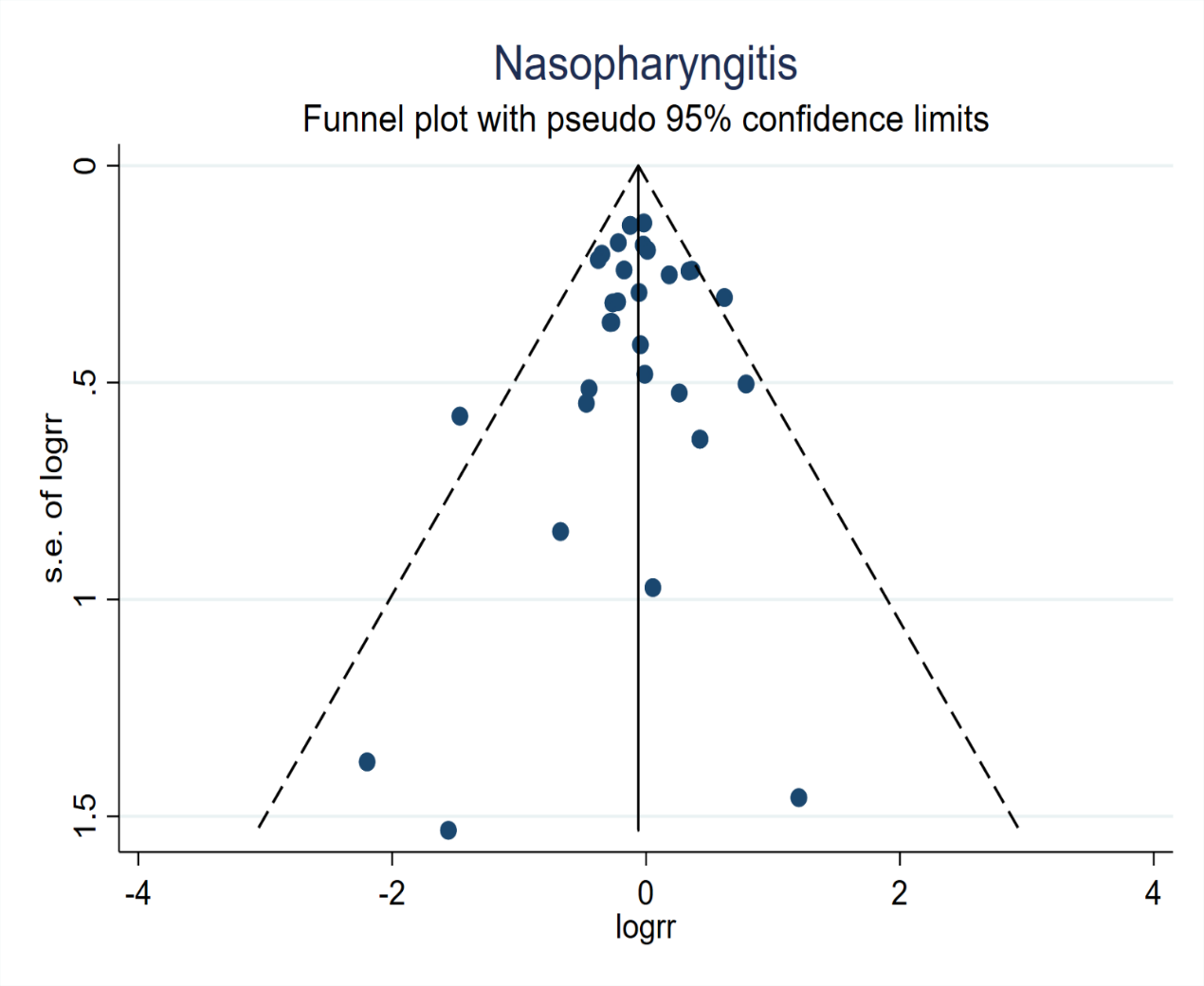
**

D.

**
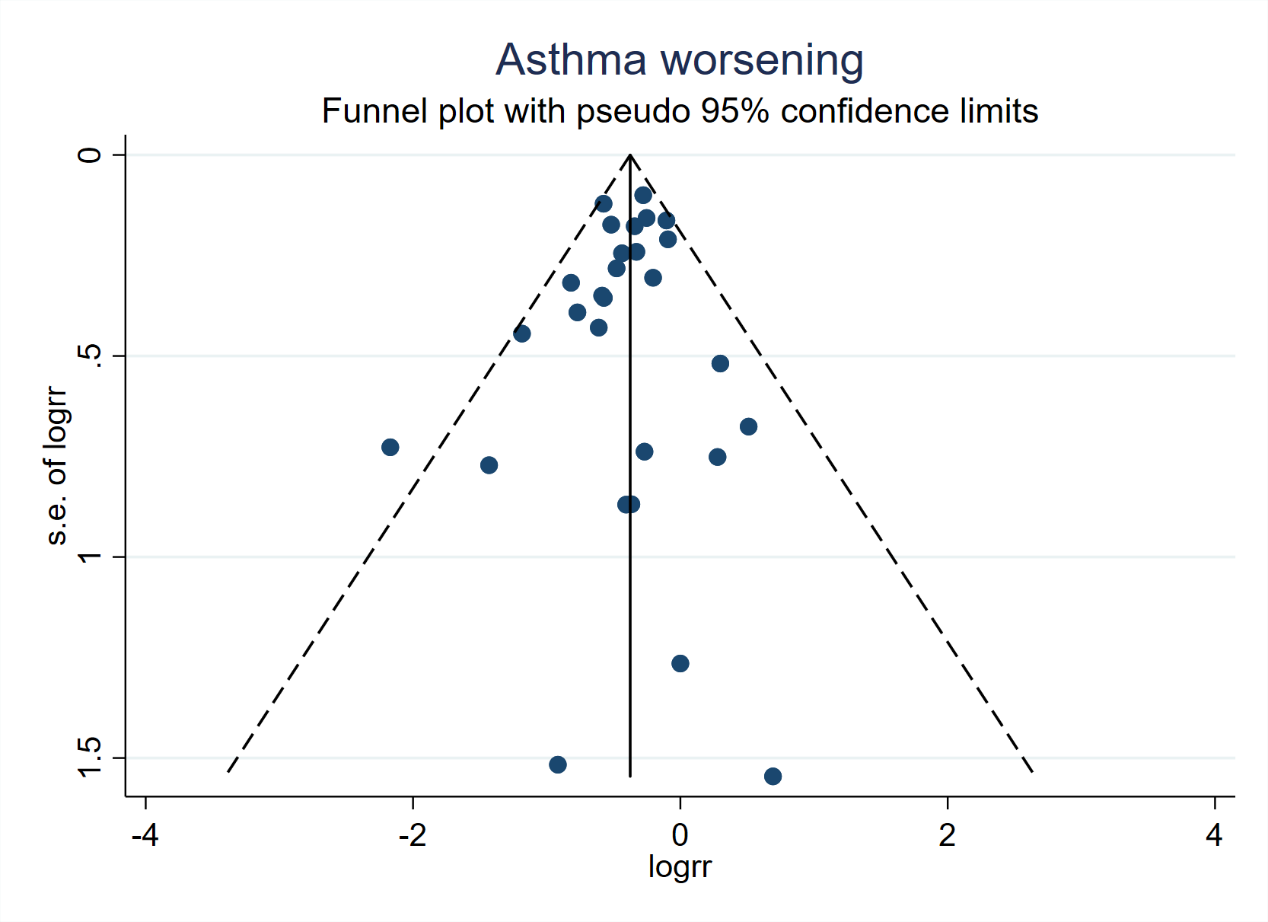
**

E.

**
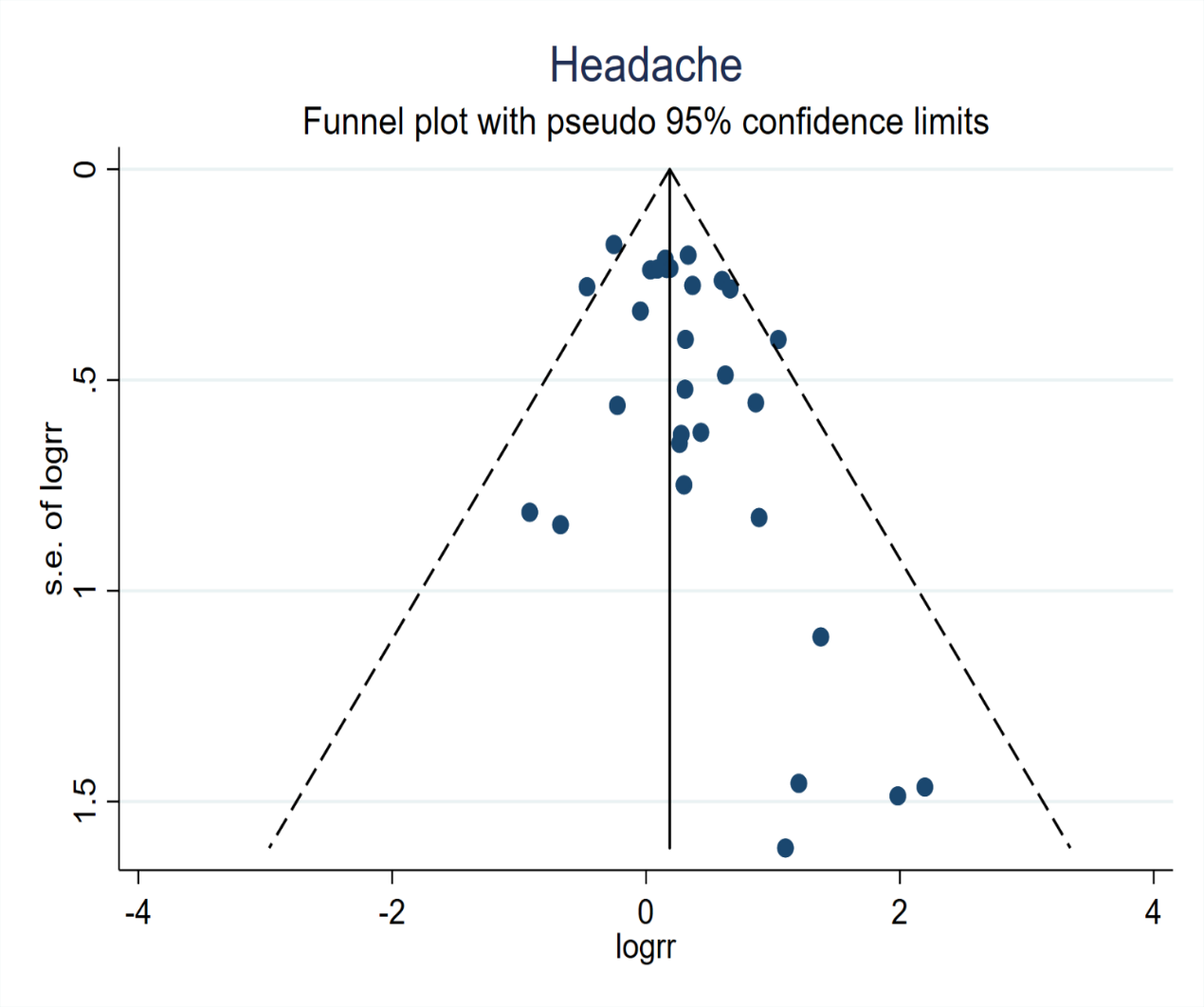
**

F.

**
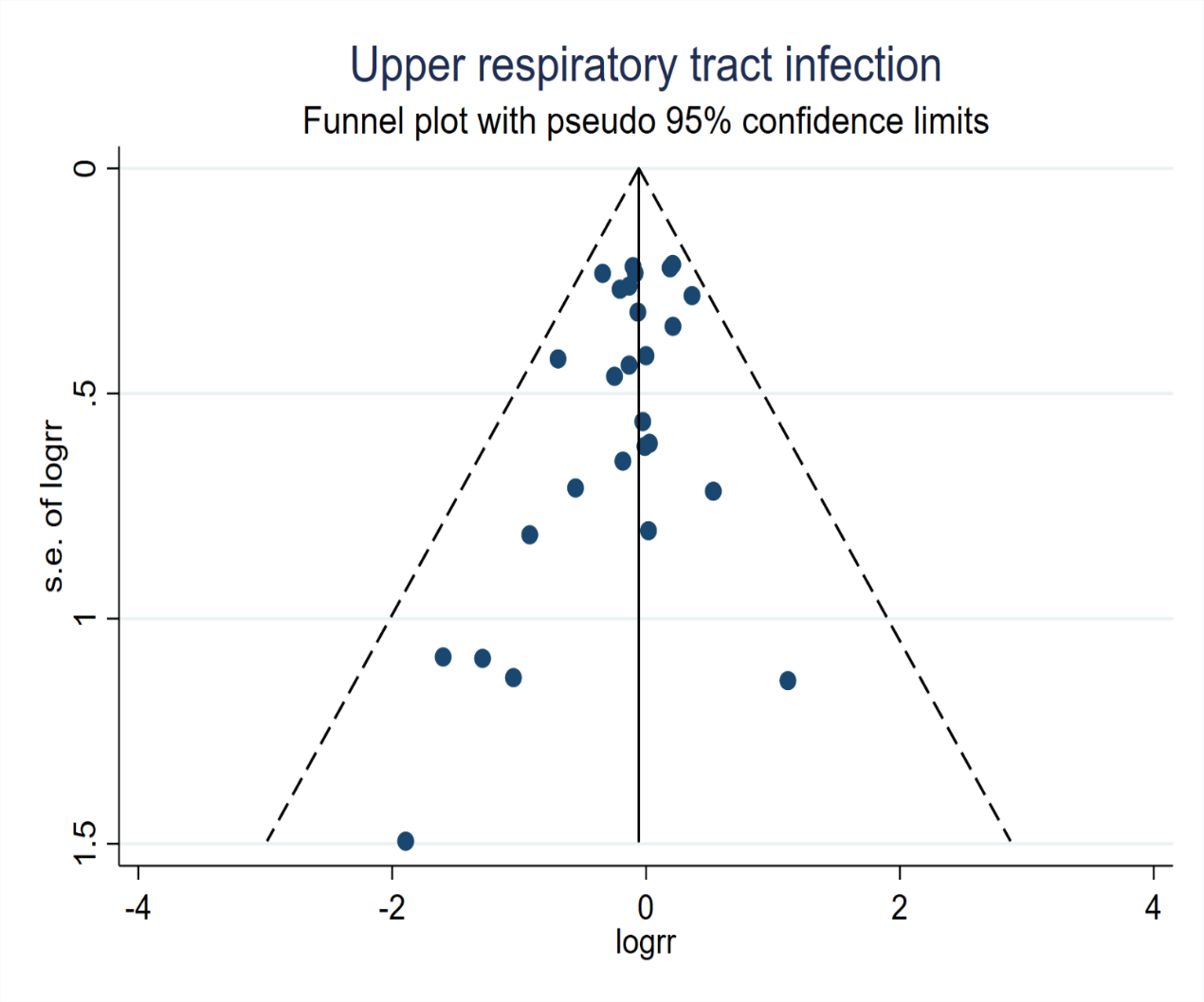
**

G.

**
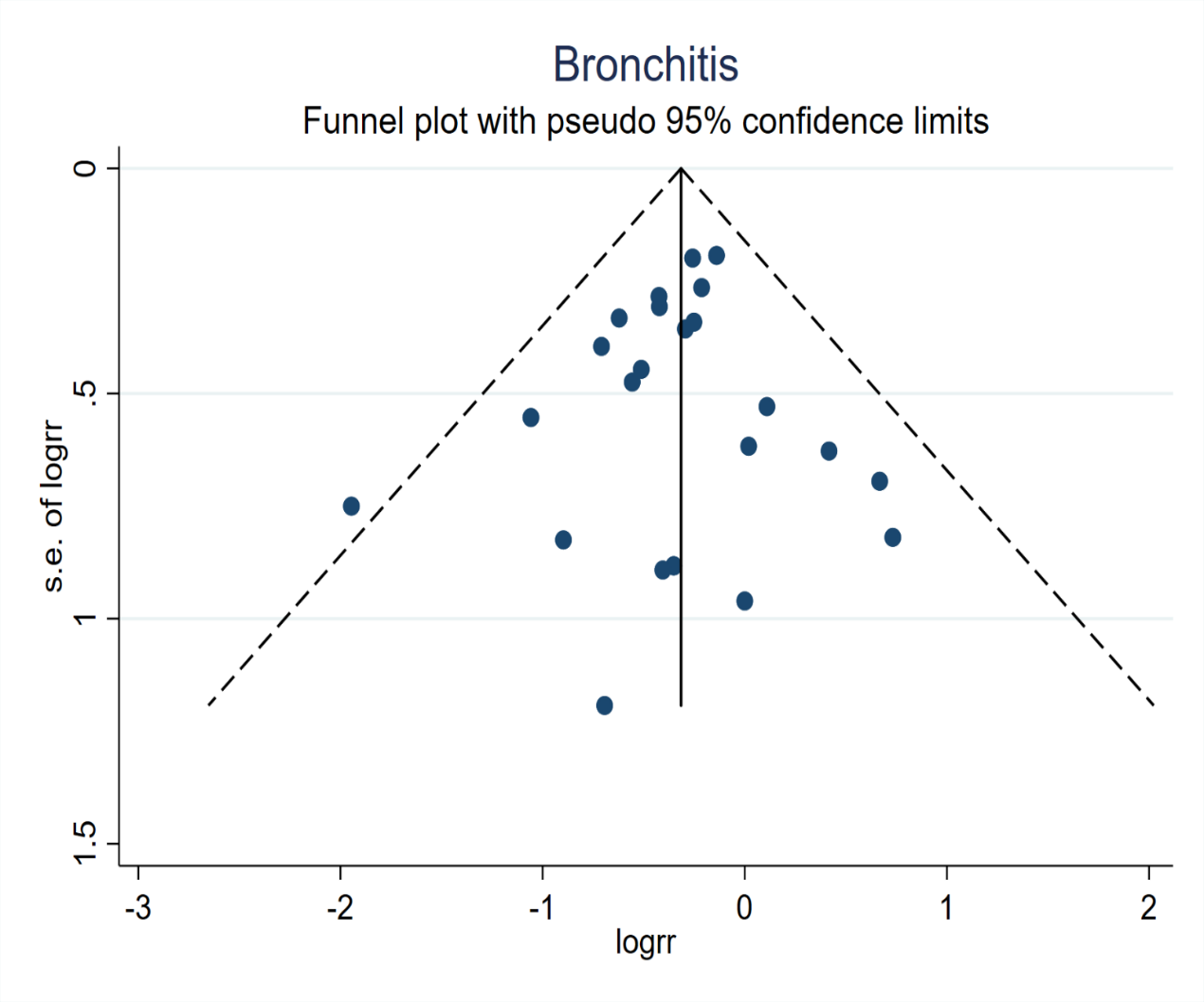
**

## **Appendix 6**

| **Secondary Results** | | | | | |
| --- | --- | --- | --- | --- | --- |
| **Variable** | **Relative Risk** | **P Value** | **Studies / Literatures (n/N)** | **Type** | **I^2^ for heterogeneity** |
| Adverse Events |  |  |  |  |  |
| Arthralgia | 1.04 (0.79-1.38) | 0.242 | 8/8 | M, B, R | 19.4% |
| Back pain | 0.93 (0.74-1.18) | 0.606 | 11/10 | M, B, R | 0.0% |
| Cough | 1.02 (0.77-1.36) | 0.494 | 8/7 | M, B, R | 0.0% |
| Diarrhoea | 0.59 (0.34-1.02) | 0.448 | 5/5 | M, B, R | 0.0% |
| Dizziness | 0.84 (0.41-1.73) | 0.078 | 6/5 | M, B, R | 45.2% |
| Fatigue | 0.97 (0.61-1.52) | 0.859 | 7/7 | M, B, R | 0.0% |
| Gastroenteritis | 1.23 (0.77-1.97) | 0.107 | 3/3 | M, B, R | 47.5% |
| Hypersensitivity adverse events^*^ | 1.06 (0.88-1.27) | 0.918 | 17/15 | M, B, R | 0.0% |
| Influenza | 0.83 (0.66-1.04) | 0.823 | 9/7 | M, B, R | 0.0% |
| Injection/Infusion site reactions^**^ | 1.89 (1.51-2.37) | 0.057 | 15/13 | M, B, R | 34.1% |
| Nausea | 0.94 (0.58-1.51) | 0.228 | 9/8 | M, B, R | 20.2% |
| Oropharyngeal pain | 0.89 (0.63-1.27) | 0.695 | 9/8 | M, B, R | 0.0% |
| Sinusitis | 0.74 (0.62-0.89) | 0.860 | 13/12 | M, B, R | 0.0% |
| Acute sinusitis | 0.77 (0.55-1.06) | 0.772 | 7/6 | B, R | 0.0% |
| Dysgeusia | 0.71 (0.01-66.40) | 0.014^b^ | 2/2 | B, R | 83.5%^a^ |
| Dyspnoea | 1.16 (0.76-1.79) | 0.214 | 6/5 | B, R | 24.9% |
| Hypertension | 0.77 (0.53-1.11) | 0.609 | 5/4 | B, R | 0.0% |
| Pharyngitis | 1.26 (0.93-1.69) | 0.672 | 6/5 | B, R | 0.0% |
| Viral gastroenteritis | 0.86 (0.09-7.80) | 0.095 | 2/2 | B, R | 64.0% ^a^ |
| Vomiting | 6.48 (1.94-21.58) | 0.996 | 3/3 | B, R | 0.0% |
| Night sweats | 1.61 (0.27-9.56) | 0.230 | 2/2 | M, B | 30.6% |
| Pain in extremity | 0.84 (0.34-2.07) | 0.054 | 2/2 | M, B | 60.7% ^a^ |
| Pyrexia | 2.24 (1.57-3.20) | 0.881 | 6/6 | M, B | 0.0% |
| Rhinitis | 1.00 (0.72-1.39) | 0.440 | 4/4 | M, B | 0.0% |
| Note: M: Mepolizumab; B: Benralizumab; R: Reslizumab; a: I^2^ for heterogeneity>50%; b: P Value<0.05  ^*^ Hypersensitivity adverse events included drug hypersensitivity to concomitant medications, food/supplement allergy, bronchospasm, urticaria, unknown allergic reaction urticaria/hives, pruritus generalised, allergy to arthropod bite, Allergic granulomatous angiitis, Rhinitis allergic, Rash, Eczema, Conjunctivitis allergic, Hypersensitivity, Angioedema, Dermatitis contact, Anti-neutrophil cytoplasmic antibody positive vasculitis, Application site rash, Dermatitis allergic, Dermatitis atopic, Drug eruption, Eosinophilic granulomatosis with polyangiitis, Immediate post-injection reaction, Injection-related reaction, Laryngospasm, Lip oedema, Rash erythematous, Rash macular, Rash maculo-papular, Rash pruritic.  ^**^ Injection/Infusion site reactions included Injection site reactions (Injection site erythema, Injection site pain, Injection site reaction) and Infusion site reactions (pain, rash, haematoma). | | | | | |

## **Appendix 7**

| **Results of Signal Detection for Adverse Events of Mepolizumab** | | | | | |
| --- | --- | --- | --- | --- | --- |
| **SOC/PT** | **Event (A)** | **PRR** | **X^2^** | **E (IC)-2SD** | **Listed** |
| **Blood And Lymphatic System Disorders** | | | | | |
| Eosinophilia^*^ | 307 | 26.00 | 7018.22 | 4.13 |  |
| **Eye Disorders** | | | | | |
| Cataract | 208 | 5.34 | 727.27 | 1.91 |  |
| **Gastrointestinal Disorders** | | | | | |
| Gastrooesophageal Reflux Disease | 333 | 6.79 | 1625.02 | 2.35 |  |
| **General Disorders And Administration Site Conditions** | | | | | |
| Adverse Drug Reaction | 274 | 4.12 | 644.72 | 1.61 |  |
| Chest Discomfort^*^ | 1,086 | 17.24 | 16105.35 | 3.83 |  |
| Chest Pain | 430 | 4.24 | 1061.12 | 1.74 |  |
| Condition Aggravated | 899 | 3.96 | 1998.20 | 1.75 |  |
| Fatigue | 1,239 | 2.31 | 938.60 | 1.01 |  |
| Gait Disturbance | 262 | 2.07 | 144.95 | 0.63 |  |
| Ill-Defined Disorder | 294 | 10.78 | 2557.18 | 2.96 |  |
| Illness | 443 | 3.20 | 669.66 | 1.34 |  |
| Injection Site Pain | 359 | 2.23 | 244.28 | 0.79 | √ |
| Malaise | 1,125 | 4.82 | 3406.73 | 2.05 |  |
| Oedema Peripheral | 247 | 4.50 | 667.84 | 1.71 |  |
| Pyrexia | 729 | 3.31 | 1181.42 | 1.47 | √ |
| Secretion Discharge^*^ | 220 | 25.41 | 4911.82 | 3.99 |  |
| Therapeutic Product Effect Incomplete^*^ | 3,032 | 30.94 | 83135.91 | 4.72 |  |
| **Immune System Disorders** | | | | | |
| Hypersensitivity | 337 | 2.47 | 295.65 | 0.93 | √ |
| **Infections And Infestations** | | | | | |
| Bronchitis | 362 | 7.92 | 2161.04 | 2.59 |  |
| Chronic Sinusitis^*^ | 198 | 92.27 | 15092.80 | 5.30 |  |
| Herpes Zoster | 226 | 5.57 | 840.54 | 1.99 |  |
| Infection | 464 | 4.69 | 1340.78 | 1.89 |  |
| Influenza | 618 | 8.26 | 3892.95 | 2.74 |  |
| Lower Respiratory Tract Infection^*^ | 617 | 19.08 | 10198.88 | 3.89 | √ |
| Nasopharyngitis | 850 | 7.07 | 4397.09 | 2.56 |  |
| Pneumonia^*^ | 2,358 | 10.80 | 20747.39 | 3.25 |  |
| Respiratory Tract Infection^*^ | 344 | 20.30 | 6072.05 | 3.85 |  |
| Sinusitis | 549 | 8.38 | 3519.81 | 2.74 |  |
| Upper Respiratory Tract Infection | 287 | 10.23 | 2344.57 | 2.89 |  |
| **Injury, Poisoning And Procedural Complications** | | | | | |
| Accidental Exposure To Product | 450 | 5.91 | 1822.19 | 2.22 |  |
| Exposure Via Skin Contact^*^ | 490 | 162.59 | 59392.97 | 6.28 |  |
| Fall | 452 | 2.08 | 256.61 | 0.74 |  |
| Inappropriate Schedule Of Product Administration | 652 | 3.25 | 1016.59 | 1.42 |  |
| Product Dose Omission Issue | 1,894 | 4.55 | 5295.55 | 2.01 |  |
| Underdose | 274 | 5.59 | 1022.74 | 2.04 |  |
| Wrong Technique In Device Usage Process^*^ | 473 | 10.76 | 4109.48 | 3.06 |  |
| **Investigations** | | | | | |
| Blood Pressure Increased | 389 | 3.68 | 758.70 | 1.52 |  |
| Blood Test Abnormal^*^ | 253 | 24.16 | 5361.13 | 3.98 |  |
| Breath Sounds Abnormal^*^ | 221 | 73.48 | 13777.21 | 5.17 |  |
| Eosinophil Count Increased^*^ | 443 | 80.35 | 29916.32 | 5.57 |  |
| Full Blood Count Abnormal^*^ | 1,855 | 77.76 | 121805.10 | 5.85 |  |
| Heart Rate Increased | 225 | 3.67 | 434.35 | 1.40 |  |
| Oxygen Saturation Decreased | 232 | 5.65 | 880.60 | 2.02 |  |
| Weight Increased | 324 | 2.26 | 227.96 | 0.79 |  |
| **Metabolism And Nutrition Disorders** | | | | | |
| Diabetes Mellitus | 220 | 5.12 | 723.00 | 1.87 |  |
| **Musculoskeletal And Connective Tissue Disorders** | | | | | |
| Arthritis | 204 | 3.85 | 427.80 | 1.45 |  |
| Back Pain | 634 | 4.25 | 1575.67 | 1.80 | √ |
| Myalgia | 287 | 2.82 | 337.41 | 1.09 |  |
| Pain In Extremity | 448 | 2.40 | 368.85 | 0.94 |  |
| **Nervous System Disorders** | | | | | |
| Headache | 1,074 | 2.65 | 1116.83 | 1.19 | √ |
| **Product Issues** | | | | | |
| Product Complaint^*^ | 515 | 16.41 | 7227.12 | 3.65 |  |
| **Psychiatric Disorders** | | | | | |
| Insomnia | 373 | 2.36 | 293.65 | 0.88 |  |
| Sleep Disorder | 230 | 4.88 | 705.07 | 1.81 |  |
| Sleep Disorder Due To A General Medical Condition^*^ | 1,724 | 246.72 | 282653.74 | 7.05 |  |
| **Respiratory, Thoracic And Mediastinal Disorders** | | | | | |
| Asthma^*^ | 6,829 | 104.70 | 582124.44 | 6.32 |  |
| Asthmatic Crisis^*^ | 454 | 211.18 | 66779.31 | 6.45 |  |
| Bronchiectasis^*^ | 305 | 70.70 | 18366.99 | 5.29 |  |
| Chronic Obstructive Pulmonary Disease^*^ | 492 | 15.62 | 6536.36 | 3.58 |  |
| Cough^*^ | 2,786 | 14.93 | 35475.06 | 3.72 |  |
| Dysphonia | 230 | 6.02 | 953.87 | 2.10 |  |
| Dyspnoea^*^ | 5,605 | 15.55 | 75349.55 | 3.81 |  |
| Dyspnoea Exertional^*^ | 675 | 24.91 | 14779.07 | 4.26 |  |
| Lung Disorder^*^ | 392 | 12.02 | 3875.30 | 3.18 |  |
| Nasal Congestion^*^ | 592 | 16.03 | 8096.95 | 3.64 | √ |
| Nasal Polyps^*^ | 316 | 160.58 | 37936.39 | 6.04 |  |
| Obstructive Airways Disorder^*^ | 1,355 | 168.50 | 168836.98 | 6.65 |  |
| Oropharyngeal Pain | 374 | 6.07 | 1569.86 | 2.22 |  |
| Productive Cough^*^ | 1,338 | 41.15 | 48512.17 | 5.02 |  |
| Pulmonary Mass^*^ | 276 | 25.12 | 6089.65 | 4.06 |  |
| Respiratory Disorder^*^ | 267 | 14.51 | 3267.23 | 3.34 |  |
| Rhinorrhoea | 415 | 8.97 | 2895.02 | 2.78 |  |
| Sputum Discoloured^*^ | 652 | 98.47 | 52573.30 | 5.91 |  |
| Upper-Airway Cough Syndrome^*^ | 276 | 52.80 | 12688.99 | 4.93 |  |
| Wheezing^*^ | 3,794 | 109.24 | 334608.56 | 6.33 |  |
| **Skin And Subcutaneous Tissue Disorders** | | | | | |
| Urticaria | 330 | 3.09 | 466.02 | 1.24 |  |
| **Social Circumstances** | | | | | |
| Loss Of Personal Independence In Daily Activities^*^ | 2,419 | 51.39 | 108649.89 | 5.37 |  |
| **Surgical And Medical Procedures** | | | | | |
| Hospitalisation | 915 | 8.21 | 5734.58 | 2.78 |  |
| **Vascular Disorders** | | | | | |
| Hypertension | 466 | 3.54 | 849.52 | 1.50 |  |
| ^*^ Indicates strong signals  √: marked as the adverse event listed in [summary of product characteristics](https://www.ema.europa.eu/en/glossary/summary-product-characteristics) | | | | | |

## **Appendix 8**

| **Results of Signal Detection for Adverse Events of Benralizumab** | | | | | |
| --- | --- | --- | --- | --- | --- |
| **SOC/PT** | **Event (A)** | **PRR** | **X^2^** | **E (IC)-2SD** | **Listed** |
| **Cardiac Disorders** | | | | | |
| Palpitations | 48 | 2.01 | 24.38 | 0.02 |  |
| Tachycardia | 42 | 2.20 | 27.37 | 0.08 |  |
| **General Disorders And Administration Site Conditions** | | | | | |
| Chest Discomfort | 200 | 9.13 | 1443.07 | 2.65 |  |
| Chills | 144 | 5.95 | 593.14 | 1.96 |  |
| Condition Aggravated | 194 | 2.51 | 178.40 | 0.83 |  |
| Death | 446 | 2.19 | 297.39 | 0.80 |  |
| Feeling Abnormal | 147 | 2.73 | 161.87 | 0.88 |  |
| Feeling Hot | 46 | 4.14 | 109.54 | 0.98 |  |
| Ill-Defined Disorder | 58 | 6.18 | 251.17 | 1.63 |  |
| Illness | 101 | 2.15 | 62.09 | 0.42 |  |
| Influenza Like Illness | 76 | 5.00 | 242.97 | 1.48 |  |
| Injection Site Pain | 172 | 3.15 | 253.99 | 1.12 | √ |
| Malaise | 235 | 2.95 | 306.05 | 1.11 |  |
| Pyrexia | 313 | 4.19 | 765.72 | 1.67 | √ |
| Swelling Face | 56 | 4.10 | 130.92 | 1.07 |  |
| Symptom Recurrence^*^ | 51 | 35.13 | 1652.35 | 3.45 |  |
| Therapeutic Product Effect Decreased | 58 | 2.54 | 54.29 | 0.44 |  |
| Therapeutic Product Effect Incomplete | 153 | 4.36 | 396.95 | 1.55 |  |
| **Immune System Disorders** | | | | | |
| Anaphylactic Reaction | 103 | 8.85 | 714.44 | 2.38 | √ |
| Eosinophilic Granulomatosis With Polyangiitis^*^ | 47 | 98.28 | 4243.80 | 4.00 |  |
| Hypersensitivity | 238 | 5.15 | 798.90 | 1.90 | √ |
| **Infections And Infestations** | | | | | |
| Bronchitis | 50 | 3.19 | 75.29 | 0.68 |  |
| Covid-19 | 177 | 2.99 | 236.68 | 1.06 |  |
| Herpes Zoster | 60 | 4.34 | 154.05 | 1.18 |  |
| Influenza | 56 | 2.18 | 35.84 | 0.21 |  |
| Nasopharyngitis | 96 | 2.33 | 73.43 | 0.52 |  |
| Pneumonia | 216 | 2.87 | 265.29 | 1.05 |  |
| Sinusitis | 60 | 2.67 | 62.63 | 0.52 |  |
| **Injury, Poisoning And Procedural Complications** | | | | | |
| Incorrect Dose Administered By Device | 99 | 14.36 | 1220.03 | 2.98 |  |
| Product Dose Omission Issue | 314 | 2.21 | 212.21 | 0.76 |  |
| Wrong Technique In Device Usage Process | 69 | 4.56 | 191.48 | 1.31 |  |
| **Investigations** | | | | | |
| Eosinophil Count Increased^*^ | 118 | 56.60 | 6209.33 | 4.61 |  |
| Full Blood Count Abnormal | 98 | 10.59 | 845.81 | 2.59 |  |
| Heart Rate Increased | 52 | 2.49 | 46.47 | 0.36 |  |
| Oxygen Saturation Decreased | 53 | 3.79 | 108.59 | 0.94 |  |
| **Musculoskeletal And Connective Tissue Disorders** | | | | | |
| Arthralgia | 245 | 2.57 | 236.98 | 0.92 |  |
| Myalgia | 125 | 3.62 | 237.91 | 1.23 |  |
| **Nervous System Disorders** | | | | | |
| Dizziness | 214 | 2.10 | 124.78 | 0.60 |  |
| Headache | 498 | 3.62 | 960.18 | 1.54 | √ |
| **Product Issues** | | | | | |
| Device Leakage^*^ | 167 | 16.72 | 2443.89 | 3.40 |  |
| Device Malfunction | 40 | 2.39 | 32.50 | 0.17 |  |
| **Psychiatric Disorders** | | | | | |
| Sleep Disorder Due To A General Medical Condition^*^ | 72 | 20.68 | 1330.81 | 3.22 |  |
| **Respiratory, Thoracic And Mediastinal Disorders** | | | | | |
| Asthma^*^ | 1,299 | 50.34 | 60983.73 | 5.35 |  |
| Chronic Obstructive Pulmonary Disease | 62 | 5.66 | 237.52 | 1.55 |  |
| Cough | 408 | 6.30 | 1829.08 | 2.29 |  |
| Dysphonia | 79 | 6.07 | 333.71 | 1.76 |  |
| Dyspnoea | 812 | 6.49 | 3821.08 | 2.44 |  |
| Dyspnoea Exertional | 56 | 5.84 | 224.27 | 1.54 |  |
| Lung Disorder | 46 | 4.09 | 107.05 | 0.96 |  |
| Nasal Congestion | 98 | 7.64 | 563.67 | 2.16 |  |
| Nasal Polyps^*^ | 40 | 46.93 | 1743.03 | 3.39 |  |
| Obstructive Airways Disorder^*^ | 82 | 22.90 | 1691.95 | 3.42 |  |
| Oropharyngeal Pain | 126 | 6.00 | 524.30 | 1.93 |  |
| Productive Cough | 102 | 8.62 | 684.20 | 2.34 |  |
| Rhinorrhoea | 47 | 2.96 | 60.92 | 0.54 |  |
| Sinus Disorder | 47 | 10.31 | 392.53 | 2.14 |  |
| Throat Tightness | 42 | 7.70 | 243.69 | 1.71 |  |
| Wheezing^*^ | 381 | 27.11 | 9425.72 | 4.29 |  |
| **Skin And Subcutaneous Tissue Disorders** | | | | | |
| Pruritus | 248 | 2.90 | 312.57 | 1.10 |  |
| Rash | 297 | 2.84 | 358.74 | 1.10 |  |
| Rash Pruritic | 63 | 5.77 | 248.05 | 1.59 |  |
| Urticaria | 239 | 6.61 | 1137.59 | 2.25 |  |
| **Social Circumstances** | | | | | |
| Insurance Issue | 42 | 13.62 | 486.83 | 2.37 |  |
| Loss Of Personal Independence In Daily Activities | 114 | 6.52 | 531.92 | 2.01 |  |
| **Vascular Disorders** | | | | | |
| Flushing | 53 | 3.03 | 72.02 | 0.63 |  |
| Hypertension | 96 | 2.15 | 58.99 | 0.40 |  |
| ^*^ Indicates strong signals  √: marked as the adverse event listed in [summary of product characteristics](https://www.ema.europa.eu/en/glossary/summary-product-characteristics) | | | | | |

## **Appendix 9**

| **Results of Signal Detection for Adverse Events of Reslizumab** | | | | | |
| --- | --- | --- | --- | --- | --- |
| **SOC/PT** | **Event (A)** | **PRR** | **X^2^** | **E (IC)-2SD** | **Listed** |
| **Endocrine Disorders** | | | | | |
| Adrenal Suppression | 8 | 1025.22 | 7907.25 | 0.88 |  |
| **Gastrointestinal Disorders** | | | | | |
| Nausea | 24 | 2.84 | 29.32 | 0.03 |  |
| **General Disorders And Administration Site Conditions** | | | | | |
| Chest Discomfort | 16 | 14.29 | 198.24 | 1.35 |  |
| Drug Ineffective | 57 | 3.32 | 96.99 | 0.73 |  |
| Injection Site Warmth | 6 | 46.43 | 266.40 | 0.09 |  |
| Paradoxical Drug Reaction | 7 | 137.87 | 946.79 | 0.55 |  |
| Therapeutic Product Effect Incomplete | 15 | 8.39 | 98.07 | 0.82 |  |
| **Immune System Disorders** | | | | | |
| Anaphylactic Reaction | 10 | 16.82 | 148.98 | 0.75 | √ |
| **Infections And Infestations** | | | | | |
| Mycobacterium Avium Complex Infection | 6 | 218.73 | 1290.78 | 0.22 |  |
| **Injury, Poisoning And Procedural Complications** | | | | | |
| Maternal Exposure During Pregnancy | 12 | 9.49 | 91.40 | 0.64 |  |
| **Investigations** | | | | | |
| Heart Rate Increased | 11 | 10.36 | 93.21 | 0.59 |  |
| **Musculoskeletal And Connective Tissue Disorders** | | | | | |
| Myalgia | 18 | 10.24 | 150.82 | 1.22 | √ |
| **Nervous System Disorders** | | | | | |
| Headache | 31 | 4.42 | 83.72 | 0.77 |  |
| **Pregnancy, Puerperium And Perinatal Conditions** | | | | | |
| Normal Newborn | 6 | 108.08 | 634.25 | 0.19 |  |
| **Respiratory, Thoracic And Mediastinal Disorders** | | | | | |
| Asthma^*^ | 62 | 45.80 | 2722.69 | 3.83 |  |
| Cough | 24 | 7.27 | 130.85 | 1.16 |  |
| Dysphonia | 9 | 13.56 | 104.81 | 0.46 |  |
| Dyspnoea | 62 | 9.71 | 492.94 | 2.18 |  |
| Oropharyngeal Pain | 19 | 17.74 | 300.82 | 1.74 |  |
| Productive Cough | 8 | 13.23 | 90.57 | 0.24 |  |
| Wheezing | 30 | 41.30 | 1180.42 | 2.92 |  |
| **Skin And Subcutaneous Tissue Disorders** | | | | | |
| Pruritus | 18 | 4.14 | 43.36 | 0.26 |  |
| Urticaria | 15 | 8.12 | 94.15 | 0.79 |  |
| **Social Circumstances** |  |  |  |  |  |
| Loss Of Personal Independence In Daily Activities | 10 | 11.21 | 93.20 | 0.50 |  |
| ^*^ Indicates strong signals  √: marked as the adverse event listed in [summary of product characteristics](https://www.ema.europa.eu/en/glossary/summary-product-characteristics) | | | | | |
